# Facing energy limitations – approaches to increase basil (*Ocimum basilicum* L.) growth and quality by different increasing light intensities emitted by a broadband LED light spectrum (400-780 nm)

**DOI:** 10.3389/fpls.2022.1055352

**Published:** 2022-11-24

**Authors:** Jenny Manuela Tabbert, David Riewe, Hartwig Schulz, Andrea Krähmer

**Affiliations:** ^1^ Julius Kühn Institute – Federal Research Centre for Cultivated Plants, Institute for Ecological Chemistry, Plant Analysis and Stored Product Protection, Berlin, Germany; ^2^ Institute of Pharmacy, Freie Universität Berlin, Berlin, Germany; ^3^ Consulting & Project Management for Medicinal and Aromatic Plants, Stahnsdorf, Germany

**Keywords:** light-emitting diode (LED), morphology, essential oil, volatile organic compound (VOC), energy use efficiency (EUE)

## Abstract

Based on the current trend towards broad-bandwidth LED light spectra for basil productions in multi-tiered controlled-environment horticulture, a recently developed white broad-bandwidth LED light spectrum (400-780 nm) including far-red wavelengths with elevated red and blue light fractions was employed to cultivate basil. Four *Ocimum basilicum* L. cultivars (cv. Anise, cv. Cinnamon, cv. Dark Opal and cv. Thai Magic) were exposed to two different rising light intensity conditions (I_Low_ and I_High_). In dependence of the individual cultivar-specific plant height increase over time, basil cultivars were exposed to light intensities increasing from ~ 100 to ~ 200 µmol m^-2^ s^-1^ under I_Low_, and from 200 to 400 µmol m^-2^ s^-1^ under I_High_ (due to the exponential light intensity increases with decreasing proximity to the LED light fixtures). Within the first experiment, basils’ morphological developments, biomass yields and time to marketability under both light conditions were investigated and the energy consumptions were determined to calculate the basils’ light use efficiencies. In detail, cultivar-dependent differences in plant height, leaf and branch pair developments over time are described. In comparison to the I_Low_ light conditions, I_High_ resulted in accelerated developments and greater yields of all basil cultivars and expedited their marketability by 3-5 days. However, exposure to light intensities above ~ 300 µmol m^-2^ s^-1^ induced light avoidance responses in the green-leafed basil cultivars cv. Anise, cv. Cinnamon and cv. Thai Magic. In contrast, I_Low_ resulted in consumer-preferred visual qualities and greater biomass efficiencies of the green-leafed basil cultivars and are discussed as a result of their ability to adapt well to low light conditions. Contrarily to the green-leafed cultivars, purple-leafed cv. Dark Opal developed insufficiently under I_Low_, but remained light-tolerant under I_High_, which is related to its high anthocyanin contents. In a second experiment, cultivars’ volatile organic compound (VOC) contents and compositions over time were investigated. While VOC contents per gram of leaf dry matter gradually decreased in purple-leafed cv. Dark Opal between seedling stage to marketability, their contents gradually increased in the green cultivars. Regardless of the light treatment applied, cultivar-specific VOC compositions changed tremendously in a developmental stage-dependent manner.

## 1 Introduction

Basil (*Ocimum basilicum* L.) is by far one of the most popular culinary herbs worldwide and is highly appreciated for its medicinal and aromatic properties. As reviewed by Filip (2017), basil essential oils have antiviral, antioxidant, anti-inflammatory, antidiabetic and antimicrobial properties. Prominent levels of phenolic acids (Jayasinghe et al., 2003; Kwee and Niemeyer, 2011), as well as substantial amounts of anthocyanins found specifically in purple basil varieties (Flanigan and Niemeyer, 2014) further contribute to basils’ potent antioxidant capacities. Besides its health-promoting properties, basil is cherished for its distinct aroma and pleasant taste. Within the species, some of the most important aroma compounds are 1,8-cineole, linalool, *ß*-caryophyllene, estragole (methyl chavicol), eugenol, methyl eugenol and methyl cinnamate (Makri and Kintzios, 2008; Schulz et al., 2003). Each compound imparts a distinct aroma (Patel et al., 2021), and their varying amounts strongly influence the basils’ flavor and consumer preferences (Walters et al., 2021).

However, the progressing climatic changes and extreme weather conditions continue to result in variable field-grown basil qualities and yields (Barut et al., 2021). To still meet the incessant consumer demand of fresh basil (Market Research Report, 2022), stakeholders are increasingly considering the implementation of environmental-friendly vertical farms and plant factories including light-emitting diodes (e.g., Kozai, 2019; Benke and Tomkins, 2018) for the production of this high-value crop. By conducting a comprehensive economic evaluation, Liaros et al. (2016) showed that basil cultivations in plant factories are principally feasible. However, the fixtures’ light intensity settings were among the most influential factors impacting its profitability. Thus, the scientific community is currently in search of minimal light intensity requirements and optimal light qualities to increase the viability of LED-based indoor basil productions.

Although lighting strategies for commercial indoor plant productions are typically adjusted during different plant stages (Currey et al., 2017) and help in reducing energy costs (Lopez and Runkle, 2008), constant (spectrum-dependent) light intensities between 180 and 545 µmol m^-2^ s^-1^ are currently recommended for indoor basil cultivations (Park et al., 2016; Sipos et al., 2021). Pennisi et al. (2020), who employed five different light intensities between 100 and 300 µmol m^-2^ s^-1^ emitted by narrow blue (B) and red (R) LEDs with main peaks at 463 and 657 nm, concluded that a constant light intensity of 250 µmol m^-2^ s^-1^ optimizes basil yield and as well as water and energy use efficiencies. However, an increase in basil yield of 20 % was recently observed when shifting the narrow B wavelength from 450 to 435 nm (Rihan et al., 2020). While basils’ optimal RB ratios are still under extensive investigation (Rihan et al., 2020; Pennisi et al., 2019; Naznin et al., 2019), inclusions of other wavelengths increasingly show great potentials for improving basil yields and qualities as well. A partial replacement of B and R with green (G) light (500-600 nm) resulted in higher basil productions by inducing stem and leaf elongations (Schenkels et al., 2020). Additions of far-red (FR) light (700-750 nm) have shown to increase basils’ canopy photosynthesis (Zhen and Bugbee, 2020), and explain the increased basil yields observed by Rahman et al. (2021) in comparison to broad white (400-700 nm) and narrow RB spectra under equal light intensity conditions. Increased fresh weights, leaf areas as well as total antioxidant capacities of basil seedlings were found under any RB spectrum that included either yellow (Y, main peak at 600 nm), G (main peak at 520 nm) or FR (main peak at 735 nm) wavelengths when compared to RB alone (Carvalho et al., 2016). However, Lin et al. (2021) demonstrated enhanced photosynthetic activities and growth of green and purple basil varieties when the proportion of R was increased in an RGB spectrum, and Song et al. (2020) showed that the inclusion of B increased the accumulation and antioxidant activity of polyphenols and essential oil content in basil when compared to different combinations of B, R and W LED light. Thus, a growing body of research suggests light spectra that consist of elevated R and B LEDs, supplemented with broad W LEDs including FR wavelengths (Sipos et al., 2021).

Based on this current trend towards broad-bandwidth LED light spectra for basil productions in controlled-environment horticulture, a white broad-bandwidth LED light spectrum (400-780 nm) with elevated R and B light fractions was developed.

It could be assumed that basil plants develop more rapidly under increasing high light conditions. Reaching market maturity earlier allows for higher plant production per cultivation unit and leads to higher profitability due to higher throughput. At the same time, basil developing under rising low light conditions with the same light composition could use light more efficiently, ultimately resulting in greater profitability due to lower energy costs. Since not only morphology but also sensory quality aspects have to be taken into account for the evaluation of profitability, the contents and compositions of essential oil (influencing aroma and flavor), which change considerably over time (i.e., Lewinsohn et al., 2000), also have to be considered.

Therefore, the aim of this study was to explore the photo-morphological development as well as the content and composition of volatile organic compounds (VOS) of four basil cultivars (cv. Anise, cv. Cinnamon, cv. Dark Opal and cv. Thai Magic) over time (from seed to common marketability) under two different rising light intensity conditions (I_Low_ and I_High_). We further aimed at investigating the basils’ biomass efficiencies under both light conditions to determine the basils’ light use efficiencies under these prototype LED light fixtures.

## 2 Materials and methods

### 2.1 Experimental design

Using a randomized block design, a three-factorial experiment (including two light intensities (I_Low_; I_High_), four basil cultivars (‘Anise’, ‘Cinnamon’, ‘Dark Opal’, ‘Thai Magic’) and four non-destructive weekly plant assessments (at 14, 21, 28 and 35 days after sowing (DAS)) with four spatially independent replications (= experimental blocks) per light treatment was conducted (*N* = 576 plant pots, *n* = 72 pots per experimental block with *n* = 18 pots per basil cultivar). To weekly collect plant material for volatile organic compound analyses, the experiment was repeated under identical conditions.

### 2.2 Plant material and growth conditions

**Figure 1:**
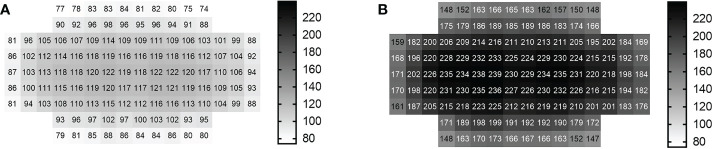
Light intensity distribution underneath each LED light treatment^1^. **(A)** Low light intensity treatment (I_Low_), **(B)** High light intensity treatment (I_High_) ^1^Each heat map depicts the photon flux densities (*PFD*s) between 400 and 780 nm [µmol m^-2^ s^-1^] measured every 100 cm^2^ within one experimental block (1.2 m^2^) underneath the light treatments at cultivation table level (*n* = 120 measurements). Gray scales to the right of each light distribution pattern are scaled from minimum to maximum *PFD*s (74-239 µmol m^-2^ s^-1^) measured across both light treatments.

Seeds of four basil cultivars, namely *Ocimum basilicum* cv. ‘Anise’, *O. basilicum* cv. ‘Cinnamon’, *O. basilicum* L. cv. ‘Dark Opal’ and *O. basilicum* L. var. *thyrsiflorum* cv. ‘Thai Magic’ were purchased from Rühlemanns’s Kräuter- und Duftpflanzen, Horstedt, Germany. In 32 seed boxes, ~ 150 seeds of each basil cultivar were sown on the surface of moist potting substrate (Fruhstorfer Einheitserde Typ P, Hawita, Vechta, Germany). Four seed boxes (each containing the seeds of one basil cultivar respectively) were placed in the center of each experimental block at equal *PFD* (photon flux density) of ~ 150 µmol m^-2^ s^-1^ and misted daily to allow for optimal light- and moisture-dependent germination. Seven days after sowing, four representative basil seedlings per cultivar were transplanted into pots (Ø = 9 cm, height 6.8 cm, volume 0.28 L) (MXC 9, Kausek, Mittenwalde, Germany). Each of the 576 pots contained a mixture of 0.28 L potting substrate (Fruhstorfer Einheitserde Typ P, Hawita, Vechta, Germany) and 560 mg of a slow-release fertilizer (15-9-11+2MgO+TE (trace elements)) (Osmocote Exact Mini 3-4M, Hermann Meyer KG, Rellingen, Germany) to ensure identical nutrient contents in all plant pots. Eight perforated propagation trays (50 x 32 x 6 cm) (CRP Import – Export GmbH, Hamburg, Germany) equipped with nine equally spaced basil pots were placed evenly under each experimental block within 1.2 m^2^ under the two different light intensity treatments. To ensure uniform watering, perforated propagation trays were submerged in unperforated propagation trays filled with equal amounts of water for 30 minutes daily. Due to inhomogeneous light intensity distributions underneath each experimental block (**Figure 1**), propagation trays were rotated and re-positioned daily to minimize positional effects. To prevent sciarid fly (*Sciardae*) infestations during experiments, nematodes (*Steinernema feltiae*) (Katz BiotechAG, Baruth, Germany) were preventively applied weekly as recommended by the supplier. At all eight experimental blocks, data loggers (EL-USB-2, Lascar, Conrad, Hirschau, Germany) continuously measured climatic conditions including temperature and relative humidity. With average temperatures (°C ± standard deviation (*SD*)) of 21.6 ± 2.8 and 21.2 ± 2.6 (with a measuring accuracy of 1°C) and average humidities (%rh ± *SD*) of 68.9 ± 13.4 and 70.8 ± 13.02 (with a measuring accuracy of 2.25%) under both light treatments, climatic conditions did not differ between light treatments.

### 2.3 Lighting systems and illumination conditions

**Figure 2:**
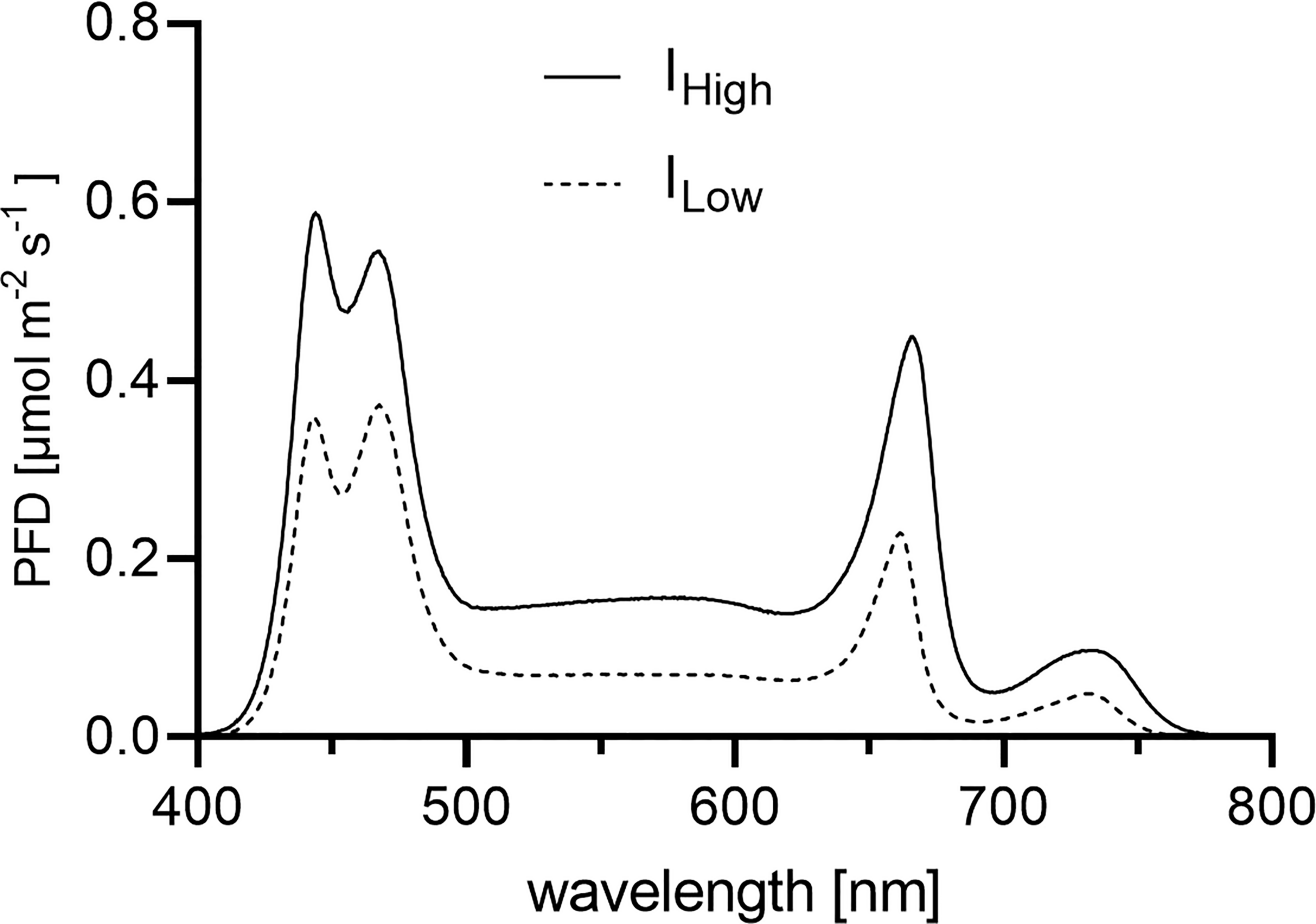
White broad-band LED light spectra applied during the experiments. Depicted are the LED light spectra as *PFD*s (photon flux densities) [µmol m^-2^ s^-1^] per wavelength [nm] between 400 and 780 nm. Each LED light spectrum (I_Low_ light treatment = dashed line; I_High_ light treatment = solid line) represents the average PFDs per wavelength obtained from 120 measurements across the entire experimental block (1.2 m^2^).

**Figure 3:**
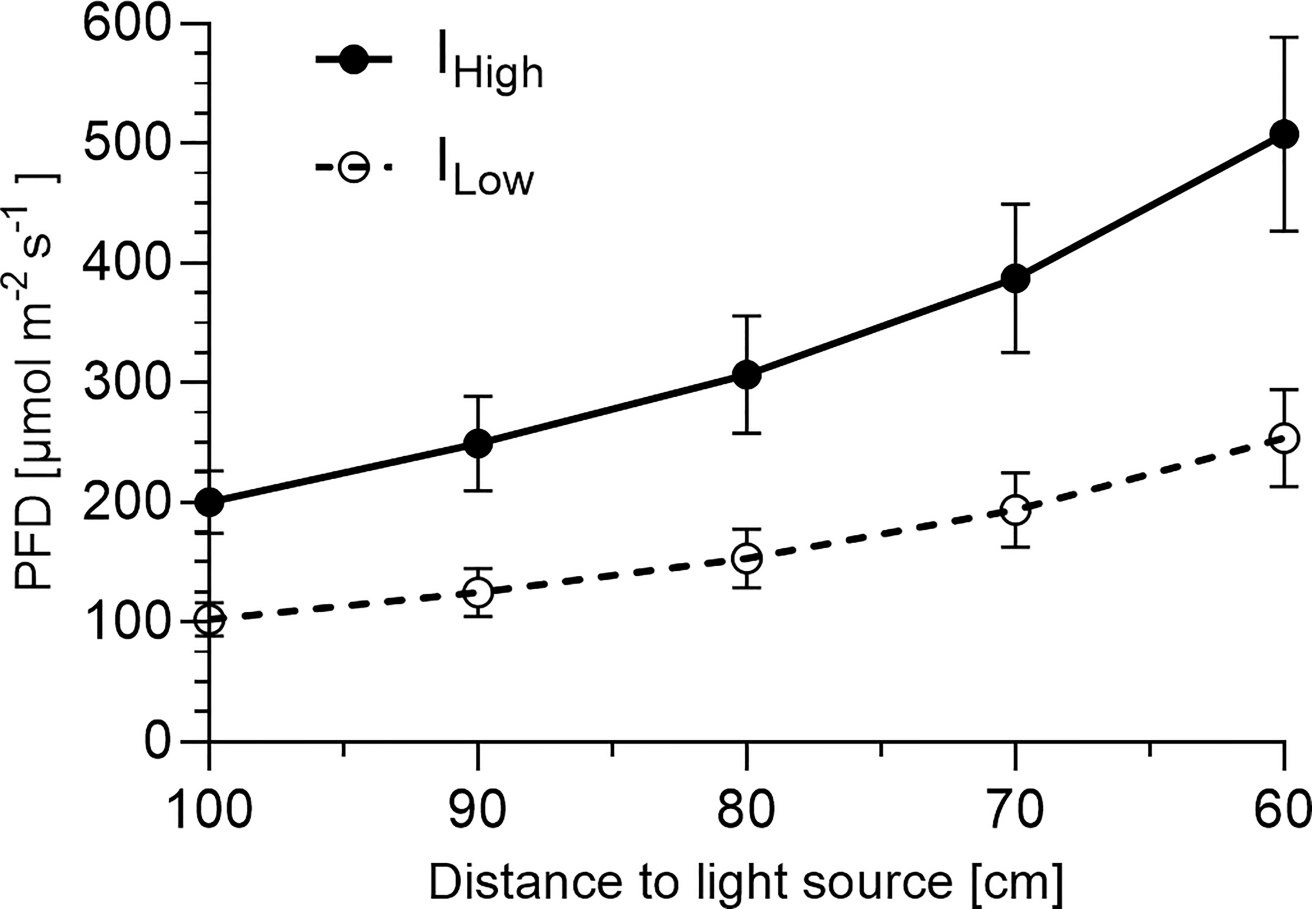
Photon flux density depending on distance to light source^1. 1^Presented are average photon flux densities (*PFD*) [µmol m^-2^ s^-1^] ± *SD* between 400 and 780 nm recorded in 10 cm intervals (starting at cultivation table level 100 cm from the light source) from nine representative positions across the cultivation area (1.2 m^2^) at each distance.

Light experiments were conducted in climate-controlled cultivation rooms. Each of the eight experimental blocks per experiment consisted of two prototype LED lamps (Apollo R1, FUTURELED^®^, Berlin, Germany) which were mounted 0.67 m apart from another and mounted onto given steel frames resulting in 1.00 m between the cultivation table and the bottom of the LED lights. The illuminated area of 1.2 m^2^ underneath these two multispectral LED lamps represented one of eight experimental blocks per experiment. Plastic sheeting extending from above the light fixtures to below the cultivation tables eliminated neighboring light pollution from other experimental blocks. Light intensity distributions, broad LED light spectra and spectral compositions during the experiments are depicted in **Figures 1**, **2** and **Table 1**, and were measured with an Ocean FX-UV-VIS spectrometer (Ocean Insight, Ostfildern, Germany). All basil seeds and seedlings were subject to a broad LED light spectrum (400-780 nm) with elevated (R) and blue (B) wavelength proportions under a *PFD* of ~ 150 µmol m^-2^ s^-1^ during germination. Starting seven days after sowing, basil plants were then subject to either a *PFD* of ~ 102 µmol^-2^ s^-1^ (= I_Low_) or a *PFD* of ~ 200 µmol m^-2^ s^-1^ (= I_High_) at cultivation table level for the remainder of the light experiments. Over time, *PFD*s increased with increasing height of basil cultivars (as the distance of the canopy to the LED light sources decreased) (**Figure 3**). Plants were subject to artificial lighting from 6.00 am to 10.00 pm for a photoperiod of 16 hours per day during the experiments (duration 35 days).

**Table 1:** Spectral compositions of the white broad-band LED light spectra^1^.

	I_Low_	I_High_
	(Photosynthetic) photon flux densities [µmol m^-2^ s^-1^]^1^	
*PFD* (400~780nm)	102 ± 14	200 ± 26
*PPFD* (400~700nm)	96 ± 13	185 ± 24
B (400~500nm)	50 ± 7	82 ± 11
G (500~600nm)	21 ± 3	44 ± 6
R (600~700nm)	25 ± 4	59 ± 8
FR (700~780nm)	6 ± 1	14 ± 3

I_Low_, Low light intensity treatment; I_High_, High light intensity treatment; PFD, Total photon flux density (400-780 nm); PPFD, photosynthetic photon flux density (400-700 nm); B, blue photons; G, green photons; R, red photons; FR, far-red photons.

^1^Presented are mean photon flux densities ± standard deviations (SD) expressed in µmol m^-2^ s^-1^ from 120 measurements taken every 100 cm^2^ across an experimental block under each light treatment at cultivation table level.

### 2.4 Energy measurements

The power draw of current (A) and voltage (V) from representative lamps of each light treatment were measured using a power meter (ENERGY MONITOR 3000, Voltcraft ^®^, Wernberg-Köblitz, Germany) to estimate the yield efficacy of the LED light treatments for each basil cultivar. Electrical consumptions were calculated by multiplying the power consumptions with the running hours of the LED light fixtures (16 hours per day, 35 days in total). Biomass efficacy (total fresh yields per kWh and cultivation area (1.2 m^2^)) as well as VOC efficacy (volatile organic compound yields per kWh and cultivation area) were calculated by multiplying the cultivars’ average yields per experimental area with 72 (= maximum number of pots per experimental block) and dividing these yields by the total kilowatt hours used per experimental block.

### 2.5 Extraction of volatiles

After samples were gently air-dried in a drying oven at 30 °C for ≤ 7 days until stable mass was attained and shortly stored under cool, dry and dark conditions, volatile compounds of basil leaves were extracted according to the following procedure: 100 mg (± 2 %) of dried and powdered (3 intervals of 10 seconds at 15,000 rpm *via* Tube Mill control, IKA^®^, Staufen, Germany) basil leaves from two basil pots (of the same cultivar, light treatment, harvest date and experimental block) were transferred into 2 mL screw cap micro tubes (Sarstedt AG & Co. KG, Nümbrecht, Germany) including two steal grinding balls (Ø 2 mm) and homogenized in 1.0 mL of high-performance liquid chromatography grade isooctane (Th. Geyer, Renningen, Germany) [containing 1:2000 (v/v) carvacrol as internal standard] for 10 minutes at 30 rps with a ball mill (MM400, Retsch^®^, Haan, Germany). After 10 minutes of ultra-sonification (Sonorex RK 106, Bandelin electronic GmbH & Co. KG, Berlin, Germany) and 10 minutes of centrifugation at 13,000 rpm (Heraeus™ Labofuge™ 400 R, Thermo Scientific™, Osterode, Germany) at 22 °C respectively, the supernatants were transferred into GC-vials and stored at -70 °C until analysis (*N* = 256 samples).

### 2.6 GC-FID and GC-MS analyses

1 µL of the obtained extracts of volatiles were analyzed by GC–FID using an Agilent gas chromatograph 6890N fitted with a HP-5MS column (30 m × 250 µm x 0.5 μm) in split mode (1:20). Detector and injector temperatures were set to 250°C. The following oven temperature program was used: 50°C for 2 min, heating from 50 to 320°C at a rate of 5°C min^–1^. The final temperature was held for 6 min. Hydrogen was used as carrier gas with a constant flow rate of 1.2 mL min^-1^. GC-MS was performed using an Agilent 5975 Network mass spectrometer, on a HP-5MS column (see GC), operating at 70 eV ionization energy, using the same temperature program as above. Helium was used as carrier gas with a constant flow rate of 1.2 mL min^-1^. Retention indices were calculated by using retention times of C_6_-C_24_-alkanes (Merck KGaA, Darmstadt, Germany) that were injected under the same chromatographic conditions.

### 2.7 Identification and quantification of volatile compounds

All main compounds of the volatile extracts were identified by comparing their mass spectra with those of internal reference libraries (Adams, NIST). Additionally, identification of 1,8-cineole, *α*-pinene, *α*-terpineol, *β*-elemene, *β*-myrcene, *β*-pinene, borneol, camphor, carvacrol, eugenol, *β*-farnesene, limonene, linalool, methyl chavicol, methyl cinnamate, methyl eugenol, ocimene and sabinene was affirmed by pure standard substances (purchased as analytical standards with a purity ≥ 95 % for GC reference analysis from Abcam (Cambridge, United Kingdom), Alfa Aesar (Kandel, Germany), Carl Roth (Karlsruhe, Germany), Fluka (Seelze, Germany), Merck KGaA (Darmstadt, Germany) and TCI GmbH (Eschborn, Germany)) and confirmed by comparing their retention indices. Volatile compounds were quantified based on the known concentration of the internal standard carvacrol.

### 2.8 Statistics and calculations

GraphPad Prism (version 9.3.1.471, San Diego, USA) was used for statistical analyses and graphical representations of all data sets. Plant heights, fresh/dry weights, VOC contents and compositions: Normality of data sets were tested *via* Shapiro-Wilk or D’Agostino & Pearson tests within and across spatial replications at α < 0.05. When distributions deviated from normality, outliers were identified *via* ROUT method at Q = 10 % and removed to obtain normality. When standard deviations (SDs) of data sets passed Brown-Forsythe and Bartlett’s tests at α < 0.05, ordinary one-way ANOVAs followed by Tukey’s multiple comparisons test at 95% confidence level (α < 0.05) were applied; when SDs were not equal between light treatments and cultivars, Brown-Forsythe and Welch ANOVAs followed by Dunnett’s T3 multiple comparisons test at 95 % confidence level (α < 0.05) were applied to analyze differences between light treatments and cultivars. (Data set of plant height: *N* = 566-575, *n* = 14-18 average plant heights per basil cultivar, light treatment and spatial replication: each *n* used for statistical analysis represents the average plant height of all four measured basil plants per pot; data sets of fresh/dry weights: *N* = 570/567, *n* = 15-18 values per cultivar, light treatment and spatial replication; data sets of volatile organic compound (VOC) content and composition: *N* = 32-64, *n* = 4-8 values per cultivar and light treatment). Number of leaf and branch pairs: As no differences within light treatments across spatial replications were observed after applying a Kruskal-Wallis test followed by Dunn’s multiple comparisons test at confidence level 95% (P < 0.05) for basil plants at each time point (*p* > 0.99), data sets of all four spatial replications per light treatment and cultivar were combined for further statistical analysis. To analyze differences between light treatments and cultivars at each time point, Kruskal-Wallis tests followed by Dunn’s multiple comparisons test at 95 % confidence level (P < 0.05) were conducted, respectively (Data set: *N* = 576, *n* = 72 assessed pots per light treatment, cultivar and time point; each *n* used for statistical analysis represents the average number of leaf pairs/side branches from all four assessed basil plants per pot). Principal component regressions (PCRs) were performed to determine the predictive power of morphological parameters (plant height, number of leaf and branch pairs) on the basils’ VOC compositions by including the average values of all morphological observations (dependent variables) and of all detected VOCs (independent variables) per light treatment and DAS. Thus, data sets of each cultivar consisted of eight average values per each of the three dependent variables (as each value represents the average morphological observation per time (14, 21, 28, 35 DAS) and light treatment (I_High_, I_Low_) and eight values per each of the 21-25 independent variables (as we detected 21, 22, 23 and 25 VOCs in cv. Anise, Dark Opal, Cinnamon and Thai Magic, respectively). Yield efficacy: The average fresh weights and leaf dry weights from 15-18 harvested basil pots per cultivar and spatial replication were multiplied by factor 72 to approximate the basil cultivars’ fresh weight and VOC productions for the entire illuminated experimental area. Approximated production yields were then divided by the calculated kilowatt hours consumed under the experimental area of 1.2 m^2^ by the end of the trial period to determine biomass and VOC efficacies. When data sets passed normality *via* Shapiro-Wilk test at α < 0.05, data sets were analyzed *via* unpaired t test, otherwise *via* non-parametric Kolmogorov-Smirnov test at 95 % confidence level (P < 0.05), respectively.

## 3 Results and discussion

### 3.1 Cultivar-dependent morphological differences

**Figure 4:**
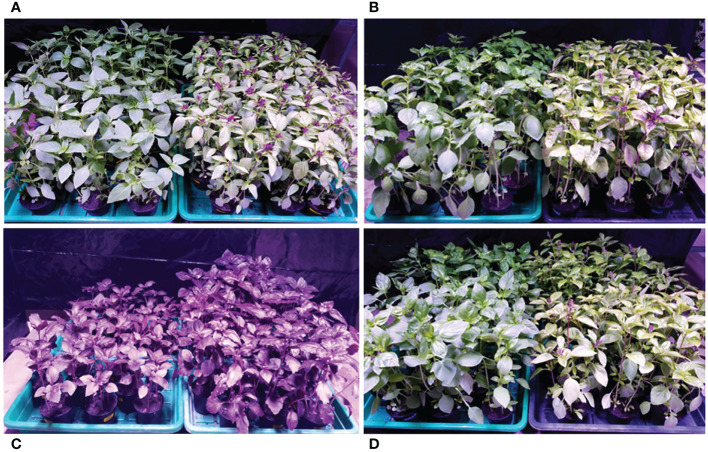
Visual appearance of four *Ocimum basilicum* L. cultivars at harvest. **(A)** *O. basilicum* L. cv. ‘Anise’, **(B)** *O. basilicum* L. cv. ‘Cinnamon’, **(C)** *O. basilicum* L. cv. ‘Dark Opal’, **(D)** *O. basilicum* L. cv. ‘Thai Magic’ under the low (left) and high (right) light intensity, respectively, 35 days after sowing.

The species *Ocimum basilicum* L. exhibits an immense variety of different cultivars and is characterized by a high intra-specific diversity in morphological traits, and the specific appearances of each cultivar observed in our study coincide with their typical phenotypes described by Carović-Stanko et al. (2011) and Lin et al. (2021). For example, basil cultivars cv. Anise, cv. Cinnamon and cv. Thai Magic were characterized by green leaves with purple stems and flowers, while basil cv. Dark Opal was characterized by the purple color of all its aerial parts and its compact growth (**Figure 4**).

**Figure 5:**
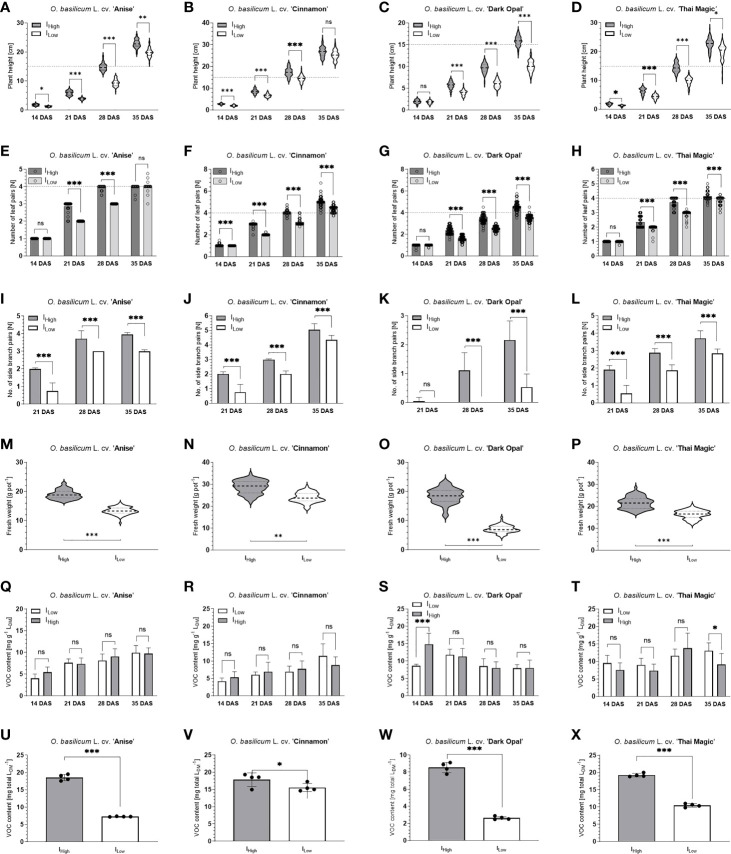
Characteristics of four Ocimum basilicum L. cultivars under two different broad-bandwidth LED light intensities over time. **A-D**: Plant height development of basil cultivar ‘Anise’ **(A)**, ‘Cinnamon’ **(B)**, ‘ Dark Opal’ **(C)** and ‘Thai Magic’ **(D)** over time (dashed and dotted lines within violin plots represent medians and quartiles (25th and 75th percentile) of the data sets, respectively; dashed lines horizontally across the graph represent marketability at height ≥ 15cm); **E-H**: Leaf pair development of basil cultivar ‘Anise’ **(E)**, ‘Cinnamon’ **(F)**, ‘ Dark Opal’ **(G)** and ‘Thai Magic’ **(H)** over time (dashed lines represent marketability at a number of leaf pairs ≥ 4); **I-L**: Branch pair development of basil cultivar ‘Anise’ **(I)**, ‘Cinnamon’ **(J)**, ‘ Dark Opal’ **(K)** and ‘Thai Magic’ **(L)** over time; **M-P**: Fresh weight per pot of basil cultivar ‘Anise’ **(M)**, ‘Cinnamon’ **(N)**, ‘ Dark Opal’ **(O)** and ‘Thai Magic’ **(P)** 35 days after sowing (at harvest; dashed and dotted lines within violin plots represent medians and quartiles (25th and 75th percentile) of the data sets, respectively); **Q-T**: Volatile organic compound content per gram of leaf dry matter of basil cultivar ‘Anise’ **(Q)**, ‘Cinnamon’ **(R)**, ‘Dark Opal’ **(S)** and ‘Thai Magic’ **(T)** over time; **U-X**: Total volatile organic compound content per total leaf dry matter of basil cultivar ‘Anise’ **(U)**, ‘Cinnamon’ **(V)**, ‘ Dark Opal’ **(W)** and ‘Thai Magic’ **(X)** 35 days after sowing (at harvest). I_High_, High light intensity treatment; I_Low_, Low light intensity treatment; DAS, days after sowing; dashed horizontal lines represent marketability (defined as /plant height ≥ 15cm and/or number of leaf pairs ≥ 4); LDM, leaf dry matter; VOC, volatile organic compound; significance levels: ns = not significant, *≤ 0.05, **≤ 0.02, ***≤ 0.01; please note the differently scaled Y-axes between cultivars.

With an average plant height of ~ 25 and ~ 27 cm and an average of four and five leaf pairs as well as branch pairs by the end of the trial period (35 days after sowing (DAS)) under I_Low_ and I_High_ light conditions, respectively, basil cv. Cinnamon grew the tallest and developed the most leaf and branch pairs from all investigated cultivars (**Table 2**; **Figure 5**). In addition, while ~ 43 % of all investigated cv. Cinnamon basil plants had developed flower buds by the end of the experiment under I_High_, no flower buds had formed under I_Low_ (**Table 2**).

**Table 2:** Summarized plant characteristics of four basil cultivars grown under two LED light intensities over time (including light intensities at plant canopy levels).

Basil cultivar		‘Anise’		‘Cinnamon’		‘Dark Opal’		‘Thai Magic’	
Parameter	DAS	I_Low_	I_High_	I_Low_	I_High_	I_Low_	I_High_	I_Low_	I_High_
Plant height [cm]^1^	14	1.2 ± 0.2^c^	1.7 ± 0.3^b^	2.0 ± 0.3^b^	2.8 ± 0.4^a^	1.8 ± 0.4^b^	2.0 ± 0.4^b^	1.2 ± 0.3^c^	1.7 ± 0.4^b^
	21	3.8 ± 0.4^c^	5.9 ± 0.8^b^	6.7 ± 0.8^b^	8.5 ± 1.0^a^	3.9 ± 0.6^c^	5.7 ± 0.8^b^	4.3 ± 0.8^c^	6.3 ± 1.1^b^
	28	9.4 ± 1.2^c^	14.8 ± 1.3^b^	14.4 ± 1.6^b^	17.4 ± 2.0^a^	6.0 ± 0.9^d^	9.6 ± 1.1^c^	9.8 ± 1.4^c^	14.5 ± 1.7^b^
	35	19.8 ± 1.4^c^	22.9 ± 1.4^b^	25.3 ± 2.2^a^	26.8 ± 2.3^a^	10.0 ± 1.3^e^	15.9 ± 1.2^d^	19.9 ± 2.5^c^	22.6 ± 2.1^b^
Light intensity at canopy level [µmol m^-2^ s^-1^]^2^	14	115 ± 17	230 ± 33	117 ± 17	236 ± 34	117 ± 17	232 ± 34	115 ± 17	230 ± 33
	21	122 ± 18	254 ± 38	130 ± 20	270 ± 40	122 ± 18	253 ± 37	123 ± 19	256 ± 38
	28	138 ± 21	312 ± 48	155 ± 24	331 ± 51	128 ± 19	276 ± 41	140 ± 21	309 ± 47
	35	175 ± 28	376 ± 60	198 ± 32	411 ± 66	140 ± 21	320 ± 49	176 ± 28	373 ± 59
Leaf pairs [n]^1^	14	1.0 ± 0.0^b^	1.0 ± 0.0^b^	1.0 ± 0.0^b^	1.0 ± 0.1^a^	1.0 ± 0.1^b^	1.0 ± 0.1^b^	1.0 ± 0.0^b^	1.0 ± 0.0^b^
	21	2.0 ± 0.0^c^	2.9 ± 0.3^a^	2.0 ± 0.1^c^	3.0 ± 0.2^a^	1.6 ± 0.3^d^	2.4 ± 0.3^b^	2.0 ± 0.2^c^	2.3 ± 0.4^b^
	28	3.0 ± 0.0^e^	4.0 ± 0.1^ab^	3.2 ± 0.3^de^	4.1 ± 0.2^a^	2.5 ± 0.3^f^	3.4 ± 0.4^cd^	2.9 ± 0.2^e^	3.7 ± 0.3^bc^
	35	4.0 ± 0.2^de^	4.0 ± 0.1^de^	4.4 ± 0.3^bc^	5.1 ± 0.4^a^	3.5 ± 0.3^f^	4.6 ± 0.4^b^	3.8 ± 0.3^e^	4.1 ± 0.3^cd^
Branch pairs [n]^1^	21	0.7 ± 0.5^b^	2.0 ± 0.1^a^	0.8 ± 0.5^b^	2.0 ± 0.1^a^	0.0 ± 0.0^c^	0.0 ± 0.1^c^	0.6 ± 0.5^b^	1.9 ± 0.2^a^
	28	3.0 ± 0.1^b^	3.7 ± 0.4^a^	2.0 ± 0.2^c^	3.0 ± 0.1^b^	0.0 ± 0.0^e^	1.1 ± 0.6^d^	1.9 ± 0.3^cd^	2.9 ± 0.2^b^
	35	3.0 ± 0.0^d^	4.0 ± 0.1^c^	4.3 ± 0.3^b^	5.0 ± 0.4^a^	0.5 ± 0.5^f^	2.2 ± 0.7^e^	2.8 ± 0.3^e^	3.7 ± 0.4^d^
Flower buds [%]^3^	35	0	100	0.0	43.1	0.0	0.0	0.0	93.1
Fresh weight [g]^1^	35	13.4 ± 1.2^e^	18.9 ± 1.6^cd^	24.0 ± 2.3^b^	28.7 ± 3.0^a^	7.1 ± 1.3^f^	18.5 ± 2.6^cd^	16.4 ± 2.0^de^	21.6 ± 2.7^bc^
Dry weight [g]^1^	35	1.2 ± 0.2^d^	3.0 ± 0.3^b^	2.1 ± 0.3^c^	3.7 ± 0.5^a^	0.5 ± 0.1^e^	1.6 ± 0.3^d^	1.5 ± 0.3^d^	3.2 ± 0.4^ab^
Leaf dry weight [g]^1^	35	0.7 ± 0.1^de^	1.9 ± 0.2^ab^	1.4 ± 0.3^bc^	2.1 ± 0.6^a^	0.3 ± 0.1^e^	1.1 ± 0.2^cd^	0.9 ± 0.2^cd^	2.2 ± 0.3^a^
VOC concentration [mg g LDM^-1^]^4^	14	4.0 ± 1.0^de^	5.4 ± 1.2^cde^	4.2 ± 0.9^e^	5.4 ± 1.6^cde^	8.6 ± 0.5^bc^	14.8 ± 3.2^a^	9.6 ± 2.2^b^	7.6 ± 2.1^bcd^
	21	7.6 ± 0.9^bc^	7.3 ± 1.4^bc^	6.1 ± 0.8^c^	6.9 ± 2.7^bc^	11.8 ± 1.6^a^	11.3 ± 2.3^a^	9.0 ± 1.9^ab^	7.4 ± 1.9^bc^
	28	8.1 ± 1.5^bc^	9.1 ± 1.8^bc^	7.0 ± 1.6^c^	7.8 ± 2.3^bc^	8.5 ± 2.2^bc^	8.0 ± 1.8^bc^	11.6 ± 1.9^ab^	13.8 ± 4.3^a^
	35	9.9 ± 1.7^ab^	9.7 ± 1.3^ab^	11.4 ± 3.5^ab^	8.8 ± 2.4^b^	7.9 ± 1.0^b^	8.0 ± 2.3^b^	13.1 ± 2.3^a^	9.1 ± 3.2^b^
VOC yield [mg total LDM^-1^]^5^	35	7.2 ± 0.1^d^	18.6 ± 0.9^a^	15.6 ± 1.2^b^	17.8 ± 2.0^a^	2.6 ± 0.2^e^	8.5 ± 0.6^cd^	10.5 ± 0.5^c^	19.2 ± 0.5^a^

DAS, days after sowing; I_Low_, Low light intensity treatment; I_High_, High light intensity treatment; VOC, volatile organic compounds; LDM, leaf dry matter; Different letters within a row indicate significant differences at P ≤ 0.05. **^1^** Presented are average values ± SD of four spatial replications (each spatial replication per basil cultivar consists of n = 14-18 plant pots, each n represents the mean value of four assessed basil plants per pot). **^2^** Presented are calculated photon flux densities (PFDs) ± SD at canopy level based on the exponential growth rate calculated in Excel via GROWTH function from the measured data points depicted in **Figure 3**. **^3^** Presented are percentage values across all four spatial replications. **^4^** Presented are average VOC contents ± SD in mg g $L_{DM}^{-1}$ (leaf dry matter) (w/w) across all four spatial replications. **^5^** Presented are calculated average total VOC quantities ± SD in mg total LDM^-1^ (leaf dry matter) across four spatial replications.

Though basil cv. Anise showed the most homogeneous development (lowest variance) of all investigated basil cultivars, cv. Anise and cv. Thai Magic developed very similarly under both LED light conditions (**Table 2**; **Figure 5**). While both cultivars reached a plant height of ~ 20 cm under I_Low_, the cultivars grew significantly taller under I_High_ and reached heights of ~ 23 cm by the end of the trial period. In addition, both cultivars developed the same number of leaf pairs along the main stem during the experiment and had developed an average of four leaf pairs along the main stem under both light conditions by the end of the experiment. Further, all cv. Anise basil plants and ~ 93 % of the cv. Thai Magic basil plants had formed flower buds under I_High_, whereas no flower bud formation was detected under I_Low_. Though branching of cv. Anise progressed faster, both cultivars averaged three and four branch pairs at the end of the trial period under I_Low_ and I_High_, respectively.

With heights of ~ 10 and ~ 16 cm, and an average of one and two branch pairs per basil plant under I_Low_ and I_High_ at harvest, respectively, the purple-leafed cv. Dark Opal remained the shortest and least branched of the four investigated cultivars (**Table 2**; **Figure 5**). Though cv. Dark Opal developed the fewest leaf pairs under the I_Low_ light conditions, its leaf pairs developed rapidly under I_High_ by growing an average of ~ 5 leaf pairs per basil plant.

### 3.2 Enhanced basil development under I_High_ results in earlier marketability than under I_Low_

All four investigated *Ocimum basilicum* L. cultivars developed more rapidly under I_High_ than under I_Low_ light conditions. During all weekly assessments, basil heights as well as the number of leaf and branch pairs were mostly greater under I_High_ when compared to the developmental stages reached under I_Low_ during the same assessment week. Further, while basil flower buds developed under I_High_ in all three green-leafed basil cultivars during the last week of the trial period, no flower bud formations were observed under I_Low_. Accordingly, all cultivars had accumulated greater total fresh and dry weights as well as leaf dry weights per basil pot under I_High_ at the end of the experiment than under I_Low_ (**Table 2**; **Figures 5A–P**).

Thus, marketability based on morphological criteria (defined as basil height ≥ 15 cm and/or number of leaf pairs along the main stem ≥ 4) of cv. Cinnamon, cv. Anise, cv. Thai Magic and cv. Dark Opal was reached 27, 29, 30 and 32 DAS under the I_High_ light treatment, respectively (**Figures 5A–H**). Though the light intensity of I_Low_ represented ~ 50 % of I_High_ (**Table 1**), green-leafed cultivars were marketable only 3-4 days later than under I_High_ conditions: While marketability of the cultivars cv. Cinnamon and cv. Thai Magic was reached three days later (30 and 33 DAS, respectively), cv. Anise reached marketability under I_Low_ four days later than under I_High_ (33 DAS). Cultivar Dark Opal did not reach the defined marketable stage under I_Low_ during the 35 days of cultivation. However, based on cv. Dark Opals’ growth function, its marketability under I_Low_ can be expected 37 DAS (5 days later than under I_High_) as the leaf pair criterium (≥ 4 leaf pairs along the main stem) should be reached.

### 3.3 I_High_ induced light avoidance responses in green basil cultivars while purple basil remained ‘light-tolerant’

As evident in **Figure 4**, undesirable morphological and anatomical leaf adaptions to I_High_ became clearly visible at the top canopy of the green-leafed cultivars cv. Anise, cv. Cinnamon and cv. Thai Magic during the last week of the trial period when PFDs exceeded an average of 300 µmol m^-2^ s^-1^ at canopy level (**Table 2**). In addition to purple pigment productions observed in some leaves of cv. Anise and cv. Cinnamon, all three green-leafed cultivars displayed leaf curling, reduced and dehydrated leaf blades and a pale green leaf color under the I_High_ light treatment, which are associated with reduced consumer preferences (Walters et al., 2021).

These leaf alterations exemplify typical light avoidance responses (Logan et al., 2015). To protect themselves from irreversible damages caused by excessive light (such as loss of oxygen evolution and loss of electron transfer activity of photosystem II (Tyystjärvi, 2013), sessile plants have evolved sophisticated signaling and protective mechanisms such as anthocyanin biosynthesis (Ma et al., 2021), chloroplast avoidance response (Wada, 2013) and thermal energy dissipation (Garcia-Plazaola et al., 2012).

The visible purple pigmentations in cv. Anise and cv. Cinnamon at the end of the trial period result from the biosynthesis of non-photosynthetic anthocyanins. Anthocyanin accumulations were proven to be stimulated by high light intensities to shield photosynthetic apparatuses from exceeding light conditions (Gould et al., 2018; Stetsenko et al., 2020; Ma et al., 2021). In example, anthocyanin contents increased by more than 20 % after LED light exposure to ≥ 300 µmol m^-2^ s^-1^ in a green-leafed lemon basil (*O. basilicum* x *O. americanum*) (Stetsenko et al., 2020), and their presence has been shown previously for both cultivars (Kwee and Niemeyer, 2011; Carović-Stanko et al., 2011). Thus, the anthocyanin formations observed in some leaves of cv. Anise and cv. Cinnamon under I_High_ (but not under I_Low_) are obviously a light-induced photoprotective mechanism.

In addition, comparative studies investigating high light responses in acyanic (green-leafed) and cyanic (purple-leafed) basil varieties (Landi et al., 2014; Tattini et al., 2014; Logan et al., 2015; Stetsenko et al., 2020) prove and support that the constitutively high anthocyanin concentrations in purple basils are responsible for mitigating light stress responses (while their green basil counterparts start to downregulate their photochemical efficiency under equal light stress conditions). Their findings explain the absence of visible photomorphogenic responses and thus, the ‘light-tolerance’ of purple-leafed ‘Dark Opal’ under I_High_ in our study, which represents a cultivar with very high anthocyanin contents (Lin et al., 2021).

The pale green color of leaves observed in all green cultivars under I_High_ is a typical sign of a chloroplast avoidance response: While the green color-giving chloroplasts distribute across the upper and lower sides of each palisade cell to achieve maximal light absorption under low to optimal light conditions (explaining the characteristic green leaf colors present under I_Low_ (**Figure 4**), chloroplasts gather at the side walls of each palisade cell to minimize the damages associated with the absorption of excessive light energy by chlorophyll pigments (Kagawa et al., 2001; Wada, 2013).

Additionally, comparable to our observations, leaves of green basil cultivars tend to reduce in size when exposed to a constant PPFD of 300 µmol m^-2^ s^-1^, which was associated with decreased chlorophyll contents, stomatal conductance, stomatal size and density in the green basil cultivar ‘Genovese’ (Pennisi et al., 2020). Reduced leaf sizes were also observed in the green cultivar ‘Tigullio’ under full sunlight when compared to leaves grown under 30 % sunlight (Tattini et al., 2014).

All described light avoidance responses of the green basil cultivars under I_High_ during the last week of the trial period are unmistakable signs of non-photochemical quenching (NPQ), a fundamental physiological photoprotective mechanism which involves the conversion and dissipation of excess excitation energy into heat. As NPQ represents a plants’ shift from a photosynthetically efficient state to a state in which a fraction of photosynthetic reaction centers is non-functional, NPQ always occurs at the expense of photosynthetic efficiency and results in limited growth and reduced crop yields (García-Plazaola et al., 2012; Logan et al., 2015).

In addition, I_High_ conditions accelerated flower initiation in green basil cultivars. Based on the seed supplier, cv. Anise, cv. Cinnamon and cv. Thai Magic are endogenously programmed to initiate flowering after eight, seven and six nodes have been developed, respectively. However, under I_High_ these cultivars initiated flowering after developing only five, six and five nodes (including cotyledons), respectively (**Table 2**; **Figure 5**), which is a phenomenon that has recently been categorized as stress-induced flowering in angiosperms (Takeno, 2016). Although stress-induced flowering has received little attention so far, previous studies of *Salvia*, *Calendula*, and *Pharbitis* have shown that intense light stress triggers an earlier flowering pulse (Moccaldi and Runkle, 2007; Hirai et al., 1994).

### 3.4 I_Low_ resulted in beneficial acclimation responses in green basil cultivars while purple basil performed insufficiently

In contrast to the undesirable light-avoiding leaf adaptations and flower initiations detected under I_High_ (section 3.3), the light-orientated leaves of the green cultivars cv. Anise, cv. Cinnamon and cv. Thai Magic were fully expanded, thin and displayed typical green leaf colors under I_Low_ (**Figure 4**), which represent decisive external quality attributes for consumers (Rouphael et al., 2012). As investigated by Walters et al. (2021), consumers prefer big, fresh basil leaves without detectable discolorations and damages as well as ‘soft’ leaf textures. Consequently, all visual traits detected under I_Low_ supersede the unsatisfactory visual attributes identified under I_High_ and translate into highly desirable basil products by meeting the consumers’ basil preferences.

These favored basil leaf qualities under I_Low_ represent acclimation responses often found in green leaves exposed to low light levels to increase light capture and photosynthetic efficiency (Nemali and van Iersel, 2004; Wada, 2013; Stagnari et al., 2018), and are to be ascribed to the great plasticity in morpho-anatomical, physiological and biochemical traits of green basils: As shown by Tattini et al. (2014), the capability of green basil cultivar ‘Tigullio’ to increase its leaf area, photosynthetic pigment concentrations and CO_2_ assimilation rates under 30% sunlight resulted in greater net photosynthesis and net daily carbon gains than detected in cv. Tigullio under full sunlight. The greater leaf sizes, the evenly distributed chloroplasts across the leaves of cv. Anise, cv. Cinnamon and cv. Thai Magic (**Figure 4**) as well as the fact that the marketability criteria were reached just 3-4 days later under I_Low_ (as described in section 3.2) are robust signs that very similar (beneficial) low light acclimation responses took place in the green cultivars investigated in our study.

To the contrary, the comparatively small leaf sizes (as evident in **Figure 4C**) and the slow development of cv. Dark Opal under I_Low_ (as evident by the great differences in plant height, number of developed leaf and branch pairs and thus biomass yields (**Table 2**, **Figures 4C, G, K, O**) in comparison to I_High_ show clearly, that cv. Dark Opal was not able to adjust as well as its green basil counterparts to the given low light conditions. The reason for cv. Dark Opals poor performance under I_Low_ can be ascribed to its high content of leaf anthocyanins (Lin et al., 2021), which proved to translate into an intrinsically low ability to maximize light interception and transmission in the similar anthocyanin-rich purple leaves of basil cultivar ‘Red Rubin’ under low light conditions (Tattini et al., 2014). The anthocyanins’ role in the observed insufficient I_Low_ performance is further strengthened by Misyura et al. (2013) who showed that anthocyanin-rich *Arabidopsis thaliana* mutants grew worse compared to the wild type under equal light conditions.

### 3.5 Cultivar-dependent light intensity requirements under increasing light intensity conditions

Providing photon fluxes that surpass the plants’ ability to absorb the supplied energy decrease photosynthetic rates and consequently result in decreasing photosynthate accumulations and biomass productions (Tyystjärvi, 2013). Hence, plants’ light saturation points (the intensity at which additional increases in light do not increase photosynthesis) should not be exceeded for optimal (and cost-effective) plant development.

The light saturation point of *Ocimum basilicum* L. was recently determined [under an RB spectrum with narrow peaks at 660 and 445 nm, ratio 7:3, utilized cultivar not provided] to be between 420 and 545 µmol m^-2^ s^-1^ (Park et al., 2016). Also Beaman et al. (2009) determined [under cool white fluorescent and incandescent lamps] high light intensity requirements of 500 µmol m^-2^ s^-1^ for optimal cultivation of green basil cultivars ‘Italian Large Leaf’, ‘Genovese’ and ‘Nufar’. In contrast, a current review by Sipos et al. (2021) concluded constant spectrum-dependent PPFDs of only 180 to 300 µmol m^-2^ s^-1^ to be adequate for basil cultivation. However, the review does not take cultivar-dependent differences into account, even though optimal light intensities are known to vary among cultivars. (Lee et al., 2019; Virŝilė et al., 2019). In our investigation, basil plants developed under rising (not constant) photon fluxes as the basils grew towards the LED light sources in proximity (**Figure 3**).

If not constantly adjusted manually, increasing light intensity gradients are common under space-limited growing conditions e.g., in vertical farms and plant factories (Tabbert et al., 2022), and could be used strategically since light requirements and the ability to absorb light energy generally increases during plant development until saturation is reached (Currey et al., 2017). For example, providing increasing light intensities resulted in shoot dry weights and leaf numbers alike those generated under constantly high light intensities (Lopez and Runkle, 2008; Oh et al., 2010) but under reduced energy costs (Poulet et al., 2014).

In general, all four of our investigated basil cultivars developed well under rising light intensity conditions which is in accordance with Solis-Toapanta and Gómez (2019), who evaluated growths and photosynthetic capacities of green basil cultivar ‘Genovese Compact’ and purple cultivar ‘Red Rubin’ under increasing and constant light intensities.

However, (as described in section 3.3) the light intensities reached under I_High_ during the last week of our experiment (28-35 DAS) led to adverse, quality-reducing effects in the investigated green basil cultivars. Average PFDs above 312, 331 and 309 µmol m^-2^ s^-1^ under I_High_ became detrimental and should therefore not be exceeded under the applied spectral composition (and growing conditions). In contrast, cv. Anise, cv. Cinnamon and cv. Thai Magic indicated no unfavorable signs under average PFDs of 175, 198 and 176 µmol m^-2^ s^-1^ under I_Low_ by the end of the experiment (**Table 2**). Hence, even under the increasing light intensity conditions applied in our study, the recommended upper limit of 300 µmol m^-2^ s^-1^ for commercial basil production under artificial lighting by Sipos et al. (2021) holds true for the investigated cultivars ‘Anise’, ‘Cinnamon’ and ‘Thai Magic’. In contrast, the light-tolerant purple basil cultivar ‘Dark Opal’ could be grown under artificial PFDs greater than 320 µmol m^-2^ s^-1^ as no obvious negative effects were detected at this light intensity during harvest, and could generate higher yields than measured in our study.

However, with the high-quality market-ready basils within 30-37 DAS (**Figures 5A–H**) detected under I_Low_ under increasing light intensities (between ~ 100 and 200 µmol m^-2^ s^-1^ (**Table 2**)) mostly below the lowest recommended constant light intensity of 180 µmol m^-2^ s^-1^ as recently reviewed by Sipos et al. (2021), the increasing light conditions applied in this study show that even with lower than previously assumed light requirements, high-quality basil productions under articificial light conditions are possible.

### 3.6 Cultivar-specific VOC concentrations change over time

The gas chromatographic analyses of basil leaf extracts resulted in the identification of 37 volatile organic compounds (VOCs), representing more than 96 % of all detected compounds. All investigated cultivars showed compositional similarities as many VOC compounds (e.g., 1,8-cineole, *ß*-elemene and methyl eugenol) were found in all four basil cultivars. Nevertheless, cultivars cv. Anise and cv. Thai Magic were characterized by high concentrations of methyl chavicol (**Tables 3**, **4**), whereas linalool, methyl chavicol, *trans*-methyl cinnamate and methyl eugenol were the major compounds of cultivar ‘Cinnamon’ (**Table 5**). Leaf extracts of the cultivar ‘Dark Opal’ were characterized by high concentrations of methyl eugenol, as well as 1,8-cineole, linalool and eugenol (**Table 6**). Thus, each cultivars’ VOC compositions as affected by the artificial light conditions applied in this study are in good agreement with published VOC compositions identified under greenhouse and field conditions (Elansary and Mahmoud, 2014; Wesolowska and Jadczak, 2016; Maggio et al., 2016; Varga et al., 2017; Dehsheikh et al., 2020).

**Table 3:** Relative abundance of major volatile organic compounds identified in the leaves of *O. basilicum* L. cv. ‘Anise’ as affected by LED light intensity treatments over time.

No.	Compound	RI^1^	I_Low_				I_High_			
			DAS 14	DAS 21	DAS 28	DAS 35	DAS 14	DAS 21	DAS 28	DAS 35
			Percent (%) of total volatile organic compound composition^2^							
1	*α*-pinene	938	–	–	0.3 ± 0.0^bc^	0.3 ± 0.0^c^	–	0.4 ± 0.0^ab^	0.4 ± 0.0^abc^	0.4 ± 0.0^a^
2	sabinene	978	–	–	0.3 ± 0.0^c^	0.3 ± 0.0^c^	–	0.4 ± 0.0^abc^	0.3 ± 0.0^bc^	0.4 ± 0.0^a^
3	*β*-pinene	982	0.9 ± 0.1^a^	0.7 ± 0.0^c^	0.7 ± 0.1^c^	0.7 ± 0.1^c^	0.9 ± 0.0^a^	0.8 ± 0.0^ab^	0.7 ± 0.1^bc^	0.9 ± 0.0^a^
4	*β*-myrcene	992	–	–	–	0.3 ± 0.0^b^	–	–	0.4 ± 0.1^b^	0.5 ± 0.0^a^
5	limonene	1033	–	–	–	0.3 ± 0.0^b^	–	–	0.3 ± 0.0^ab^	0.3 ± 0.0^a^
6	**1,8-cineole**	**1036**	**8.2 ± 0.5^bc^**	**7.0 ± 0.4^d^**	**6.8 ± 0.5^d^**	**7.2 ± 0.5^cd^**	**8.4 ± 0.2^b^**	**8.2 ± 0.3^b^**	**7.9 ± 0.8^bc^**	**9.3 ± 0.3^a^**
7	*trans*-*ß*-ocimene	1050	–	0.5 ± 0.0^d^	0.7 ± 0.1^c^	0.9 ± 0.1^b^	–	0.7 ± 0.1^c^	1.0 ± 0.1^b^	1.3 ± 0.1^a^
8	camphor	1154	1.5 ± 0.1^a^	1.3 ± 0.1^b^	1.3 ± 0.1^b^	1.4 ± 0.1^ab^	1.4 ± 0.0^ab^	1.5 ± 0.1^a^	1.5 ± 0.2^a^	1.3 ± 0.1^b^
9	*α*-terpineol	1197	–	0.7 ± 0.0^cd^	0.7 ± 0.0^d^	0.8 ± 0.0^bc^	0.8 ± 0.0^bc^	0.8 ± 0.0^b^	0.8 ± 0.1^b^	1.0 ± 0.1^a^
10	**methyl chavicol**	**1204**	**76.9 ± 1.4^abc^**	**78.5 ± 0.7^a^**	**77.4 ± 1.0^ab^**	**74.5 ± 1.2^cd^**	**76.2 ± 1.1^bc^**	**75.4 ± 1.1^cd^**	**74.0 ± 1.9^d^**	**68.9 ± 0.8^e^**
11	*cis-*methyl cinnamate	1294	–	–	–	0.3 ± 0.1^b^	–	–	0.4 ± 0.1^b^	0.5 ± 0.1^a^
12	*trans*-methyl cinnamate	1390	0.9 ± 0.1^a^	0.5 ± 0.0^c^	0.4 ± 0.0^cd^	0.3 ± 0.0^de^	0.6 ± 0.0^b^	0.4 ± 0.0^cde^	0.3 ± 0.0^e^	0.3 ± 0.0^e^
13	*β*-elemene	1402	–	–	–	0.4 ± 0.1^ab^	–	–	0.4 ± 0.1^b^	0.5 ± 0.0^a^
14	methyl eugenol	1407	–	0.5 ± 0.0^c^	0.5 ± 0.0^c^	0.5 ± 0.0^c^	0.7 ± 0.0^a^	0.6 ± 0.0^b^	0.6 ± 0.1^abc^	0.5 ± 0.0^c^
15	aromadendrene	1446	2.4 ± 0.4^abcde^	1.6 ± 0.2^bcd^	1.4 ± 0.1^de^	1.3 ± 0.2^e^	2.1 ± 0.1^a^	1.8 ± 0.1^b^	1.4 ± 0.1^de^	0.9 ± 0.1^f^
16	*β*-farnesene	1461	6.2 ± 0.8^a^	4.7 ± 0.3^b^	4.2 ± 0.5^bc^	3.4 ± 0.3^de^	4.6 ± 0.2^b^	4.0 ± 0.2^cd^	3.3 ± 0.4^e^	2.2 ± 0.3^f^
17	*α*-amorphene	1471	1.6 ± 0.2^ab^	1.1 ± 0.1^abc^	0.9 ± 0.0^bcd^	0.9 ± 0.1^cd^	1.2 ± 0.1^a^	0.9 ± 0.1^bcd^	0.9 ± 0.1^bcd^	0.8 ± 0.1^d^
18	bicyclogermacrene	1498	2.6 ± 0.5^bcd^	2.1 ± 0.2^d^	2.8 ± 0.3^c^	3.6 ± 0.2^b^	2.1 ± 0.1^d^	2.5 ± 0.4^cd^	3.7 ± 0.5^b^	4.4 ± 0.2^a^
19	*δ*-cadinene^3^	1521	–	–	–	0.3 ± 0.0^b^	–	–	–	0.5 ± 0.1^a^
20	*cis*-nerolidol	1529	–	0.4 ± 0.0^d^	0.7 ± 0.1^c^	1.0 ± 0.1^b^	–	0.7 ± 0.1^c^	1.1 ± 0.2^b^	1.5 ± 0.1^a^
21	*α*-cadinol	1657	–	0.6 ± 0.1^d^	1.2 ± 0.1^c^	1.8 ± 0.1^b^	0.6 ± 0.0^d^	1.2 ± 0.2^c^	2.1 ± 0.4^ab^	2.6 ± 0.2^a^
	TOTAL [%]^4^		100 ± 0.0	100.0 ± 0.0	100.0 ± 0.0	99.8 ± 0.2	99.8 ± 0.2	99.7 ± 0.2	99.8 ± 0.2	98.6 ± 0.3
	VOC content [%]^5^		0.4 ± 0.1^e^	0.8 ± 0.1^bcd^	0.8 ± 0.1^abc^	1.0 ± 0.2^a^	0.5 ± 0.1^de^	0.7 ± 0.1^cd^	0.9 ± 0.2^abc^	1.0 ± 0.1^ab^
	VOC content [mg total LDM^-1^]^6^					7.3 ± 0.1^b^				18.6 ± 0.9^a^

RI = retention index; DAS = days after sowing; bold compounds represent the major volatile compounds of the cultivar; different letters within a row indicate significant differences at p ≤ 0.05. **^1^** Presented are mean RIs of samples under GC-FID conditions on an HP-5MS column relative to a series of n-alkanes. **^2^** Presented are mean percentages ± SD of volatile organic compounds of all analyzed basil leaf extracts (n = 8 leaf extracts per light treatment and DAS). **^3^** Data set was analyzed via unpaired t-test with p-value ≤ 0.05. **^4^** Percentage [%] ± SD represents total of listed volatile organic compounds. (Traces of eight identified compounds (namely cis-sabinene hydrate (RI 1072), borneol (RI 1174), chavicol (RI 1253), eugenol (RI1364), α-humulene (RI 1452), germacrene D (RI 1480), spathulenol (RI 1578) epi-α-cadinol (RI 1633) are excluded from the table. **^5^** Presented are average VOC contents ± SD in percent [%] per gram of leaf dry matter (w/w) from four independent experimental replications per light treatment. **^6^** Presented are calculated average total VOC contents ± SD in mg total LDM^-1^ (leaf dry matter) across four spatial replications at harvest (35 DAS) per basil pot.

**Table 4:** Relative abundance of major volatile organic compounds identified in the leaves of *O. basilicum* L. cv. ‘Thai Magic’ as affected by LED light intensity treatments over time.

No.	Compound	RI^1^	I_Low_				I_High_			
			DAS 14	DAS 21	DAS 28	DAS 35	DAS 14	DAS 21	DAS 28	DAS 35
			Percent (%) of total volatile organic compound composition^2^							
1	*α*-pinene	938	–	–	0.3±0.0	0.2±0.0	–	–	0.2±0.0	0.2±0.0
2	*β*-pinene	982	–	0.4±0.0	0.3±0.0	0.3±0.0	–	0.4±0.0	0.4±0.1	0.4±0.0
3	*β*-myrcene	992	–	–	0.3±0.0	0.3±0.0	–	–	0.4±0.1	0.3±0.0
4	limonene	1033	0.7±0.2^abc^	0.7±0.1^a^	0.6±0.0^ab^	0.5±0.1^bc^	0.7±0.0^a^	0.7±0.1^ab^	0.6±0.1^abc^	0.5±0.0^c^
5	1,8-cineole	1036	2.5±0.5^d^	3.4±0.4^c^	3.4±0.4^c^	3.6±0.3^bc^	3.9±0.5^abc^	4.4±0.5^a^	4.2±0.6^ab^	3.9±0.4^abc^
6	*trans*-*ß*-ocimene	1050	0.7±0.1^e^	0.8±0.1^de^	1.1±0.1^c^	1.4±0.2^b^	1.0±0.1^c^	0.9±0.1^cd^	1.7±0.3^ab^	1.8±0.2^a^
7	terpinolene	1095	2.9±0.9^a^	2.4±0.6^a^	1.8±0.2^b^	1.2±0.3^c^	3.1±0.8^a^	2.2±0.6^a^	1.3±0.3^c^	0.8±0.1^c^
8	linalool	1100	–	0.4±0.1^c^	0.6±0.1^bc^	0.9±0.3^ab^	–	1.2±0.8^abc^	1.3±0.4^a^	1.2±0.3^a^
9	camphor	1154	1.1±0.1^c^	1.3±0.3^bc^	1.3±0.1^bc^	1.5±0.2^b^	1.7±0.4^ab^	2.0±0.3^a^	1.9±0.2^a^	1.4±0.2^bc^
10	borneol	1174	–	0.4±0.1	0.5±0.1	0.7±0.1	–	0.5±0.1	0.8±0.2	1.4±0.3
11	*α*-terpineol	1197	0.4±0.0^c^	0.4±0.0^c^	0.5±0.1^bc^	0.5±0.0^b^	0.5±0.1^bc^	0.5±0.0^b^	0.7±0.1^a^	0.7±0.1^a^
**12**	**methyl chavicol**	**1204**	**69.7±1.9^abcde^**	**73.0±2.6^abc^**	**73.5±2.0^a^**	**71.7±1.1^abcd^**	**68.7±4.2^cde^**	**68.6±1.8^de^**	**66.4±3.3^e^**	**70.1±2.3^abcde^**
13	*trans*-methyl cinnamate	1390	1.1±0.1^a^	0.7±0.1^b^	0.6±0.1^c^	0.3±0.0^de^	0.8±0.1^b^	0.4±0.1^cd^	0.2±0.0^e^	–
14	*β*-elemene	1403	–	–	0.3±0.0^b^	0.4±0.1^b^	–	–	0.4±0.1^b^	0.7±0.1^a^
15	methyl eugenol	1406	7.2±1.8^a^	3.0±0.4^bc^	2.2±0.6^cd^	1.4±0.2^d^	4.2±0.6^ab^	2.0±0.4^d^	1.1±0.1^e^	0.9±0.2^e^
16	*α-trans*-bergamotene	1437								
17	aromadendrene	1446	4.6±0.1^b^	4.4±0.8^b^	4.3±0.3^b^	5.2±0.8^ab^	5.4±0.5^ab^	5.7±0.8^ab^	6.5±1.1^a^	5.3±1.1^ab^
18	*β*-farnesene	1460	3.8±0.7^a^	2.6±0.3^b^	2.6±0.4^b^	2.2±0.3^bc^	3.5±0.6^a^	2.1±0.3^bc^	2.0±0.5^bc^	1.6±0.4^c^
19	bicyclogermacrene	1497	1.0±0.2^de^	0.8±0.0^e^	1.1±0.2^bcd^	1.4±0.1^ab^	1.2±0.2^bcde^	1.1±0.2^cde^	1.6±0.4^abc^	1.8±0.3^a^
20	*γ*-cadinene	1514	–	–	0.3±0.1^c^	0.4±0.1^bc^	–	–	0.5±0.1^ab^	0.5±0.1^a^
21	*δ*-cadinene	1521	–	–	0.3±0.1^c^	0.5±0.2^bc^	–	–	0.7±0.1^ab^	0.9±0.2^a^
22	*cis*-nerolidol	1529	–	0.4±0.0^d^	0.6±0.1^c^	0.8±0.1^b^	–	0.8±0.2^bc^	1.1±0.2^ab^	1.3±0.2^a^
23	spathulenol	1578	0.5±0.1^c^	1.2±0.5^ab^	0.7±0.4^bc^	0.6±0.2^bc^	1.0±0.2^b^	2.0±0.5^a^	0.6±0.2^bc^	0.6±0.1^bc^
24	*epi*-*α*-cadinol	1633	–	–	–	0.3±0.0^b^	–	–	0.3±0.0^a^	0.4±0.1^a^
25	*α*-cadinol	1657	0.4±0.1^f^	0.7±0.1^e^	1.1±0.2^cd^	1.5±0.2^bc^	0.8±0.2^def^	1.3±0.3^bcd^	1.9±0.4^ab^	2.3±0.3^a^
	TOTAL [%]^3^		96.7±0.7	97.5±0.6	97.9±0.4	97.6±0.4	97.2±0.6	96.6±0.2	96.5±1.1	97.6±0.9
	VOC content [%]^4^		0.9±0.1^bc^	0.9±0.2^bc^	1.2±0.2^abc^	1.3±0.2^ab^	0.8±0.2^c^	0.7±0.2^c^	1.4±0.4^a^	0.9±0.3^bc^
	VOC content [mg total LDM^-1^]^5^					10.5±0.5^b^				19.2±0.5^a^

RI = retention index; DAS = days after sowing; the bold compound represents the major volatile compound of the cultivar; different letters within a row indicate significant differences at p ≤ 0.05. **^1^** Presented are mean RIs of samples under GC-FID conditions on an HP-5MS column relative to a series of n-alkanes. **^2^** Presented are mean percentages ± SD of volatile organic compounds of all analyzed basil leaf extracts (n = 8 leaf extracts per light treatment and DAS). **^3^** Percentage [%] ± SD represents total of listed volatile organic compounds. (Traces of nine identified compounds (namely camphene (RI 954), sabinene (RI 978), cis-sabinene hydrate (RI 1072), chavicol (RI 1253), nerol (RI 1227), cis-methyl cinnamate (RI 1294), eugenol (RI 1364), α-humulene (RI 1452), α-amorphene (RI 1471), germacrene D (RI 1479), trans-cadina-1,4-diene (RI 1534), viridiflorol (RI 1596) and traces of four unidentified compounds (RI 1015, RI 1120, RI 1261, RI 1557) are excluded from the table.) **^4^** Presented are average VOC contents ± SD in percent [%] per gram of leaf dry matter (w/w) from four independent experimental replications per light treatment. **^5^** Presented are calculated average total VOC contents ± SD in mg total LDM^-1^ (leaf dry matter) across four spatial replications at harvest (35 DAS) per basil pot.

**Table 5:** Relative abundance of major volatile organic compounds identified in the leaves of *O. basilicum* L. cv. ‘Cinnamon’ as affected by LED light intensity treatments over time.

No.	Compound	RI^1^	I_Low_				I_High_			
			DAS 14	DAS 21	DAS 28	DAS 35	DAS 14	DAS 21	DAS 28	DAS 35
			Percent (%) of total volatile organic compound composition^2^							
1	1,8-cineole	1036	3.0±0.7^ab^	2.5±0.6^ab^	2.4±0.8^b^	3.4±0.7^a^	3.3±0.7^ab^	3.1±0.5^ab^	2.7±0.4^ab^	2.6±0.6^ab^
2	*trans*-*ß*-ocimene	1049	0.6±0.0	0.6±0.1	0.5±0.2	0.5±0.1	0.6±0.3	0.7±0.1	0.5±0.0	0.6±0.3
3	terpinolene	1094	1.6±0.4^a^	0.9±0.3^bc^	0.6±0.2^bc^	0.4±0.1^c^	1.6±0.4^a^	1.0±0.4^b^	0.5±0.1^bc^	0.5±0.1^bc^
**4**	**linalool**	**1101**	**7.9±2.2^e^**	**13.8±2.5^d^**	**18.8±1.8^c^**	**23.7±3.1^ab^**	**13.6±3.0^d^**	**20.1±2.8^bc^**	**27.2±3.1^a^**	**23.5±5.0^abc^**
5	camphor	1153	0.8±0.1	0.6±0.1	0.6±0.2	0.5±0.2	0.8±0.1	0.8±0.3	0.5±0.2	0.5±0.2
6	*α*-terpineol	1184	0.9±0.1	0.8±0.2	0.9±0.3	0.7±0.2	0.7±0.1	0.9±0.2	0.7±0.3	0.7±0.5
**7**	**methyl chavicol**	**1203**	**26.8±11.3^a^**	**20.5±5.4^ab^**	**15.3±7.1^bc^**	**8.7±5.0^c^**	**17.7±10.0^abc^**	**12.0±4.8^bc^**	**9.1±6.5^bc^**	**9.8±4.2^bc^**
9	eugenol	1364	1.7±1.1	1.6±0.5	3.1±1.6	5.2±4.8	3.8±2.2	1.5±0.7	5.9±5.1	2.1±2.5
10	***trans*-methyl cinnamate**	**1391**	**8.7±3.8^d^**	**30.2±6.0^abc^**	**35.1±8.2^a^**	**33.3±8.4^a^**	**20.2±6.4^bcd^**	**34.4±6.2^a^**	**27.5±5.6^ab^**	**36.9±12.1^a^**
**11**	*β*-elemene	1403	0.7±0.0	0.5±0.1	0.6±0.2	0.9±0.3	0.4±0.0	0.6±0.2	0.7±0.2	0.9±0.3
12	**methyl eugenol**	**1406**	**27.5±10.8^a^**	**13.2±3.7^abc^**	**6.7±1.9^def^**	**4.0±2.9^efg^**	**18.2±8.9^abcd^**	**7.5±1.6^cde^**	**3.3±2.2^fg^**	**0.9±0.2^g^**
13	aromadendrene	1446	2.2±0.6^abc^	1.4±0.5^abcd^	1.3±0.2^d^	1.3±0.4^cd^	2.3±0.6^ab^	1.9±1.1^bcd^	2.0±1.2^bcd^	0.9±0.3^d^
14	*α*-humulene	1451	–	–	0.4±0.1^b^	0.6±0.2^ab^	–	0.4±0.0^b^	0.8±0.3^a^	0.7±0.2^ab^
15	*β*-farnesene	1460	5.6±0.7^b^	4.3±0.5^c^	3.0±0.5^de^	2.2±0.5^ef^	6.7±1.0^a^	3.5±0.5^cd^	2.5±0.5^def^	1.6±0.5^f^
16	*α*-amorphene	1471	3.1±1.0^a^	1.2±0.3^bc^	0.8±0.1^d^	–	1.6±0.3^ab^	1.0±0.2^cd^	1.0±0.0^c^	0.6±0.2^d^
17	germacrene D	1479	–	–	–	0.4±0.0^b^	–	0.3±0.0^c^	0.5±0.0^a^	0.4±0.1^ab^
18	bicyclogermacrene	1497	4.3±0.6^ab^	3.6±0.3^b^	3.8±0.2^ab^	4.3±0.3^a^	4.0 ±0.5^ab^	3.8±0.2^ab^	5.0±0.7^a^	4.9±0.7^ab^
19	*γ*-cadinene	1513	0.8±0.2^b^	1.0±0.2^ab^	1.1±0.1^ab^	1.4±0.2^a^	1.1±0.2^ab^	1.2±0.1^ab^	1.6±0.6^ab^	1.5±0.5^ab^
20	*δ*-cadinene	1521	–	0.5±0.0^b^	0.5±0.3^b^	1.0±0.4^ab^	–	0.6±0.1^b^	1.3±0.3^a^	1.0±0.3^ab^
21	*cis*-nerolidol	1529	1.3±0.3^c^	1.3±0.1^c^	1.6±0.2^bc^	2.1±0.2^a^	1.4±0.2^c^	1.8±0.3^b^	2.5±0.1^a^	2.5±0.4^a^
22	*epi*-*α*-cadinol	1633	–	–	0.4±0.1^c^	0.6±0.1^bc^	–	0.5±0.1^c^	0.7±0.1^ab^	0.7±0.1^a^
23	*α*-cadinol	1657	2.4±0.6^d^	2.5±0.2^d^	3.2±0.6^cd^	4.2±0.5^ab^	2.6±0.7^d^	3.4±0.8^bcd^	5.1±0.8^a^	5.1±1.0^ab^
	TOTAL [%]^3^		99.6±0.5	100.0±0.0	99.7±0.5	98.4±0.6	99.6±0.7	99.2±0.8	98.6±0.6	97.8±1.0
	VOC content [%]^4^		0.4±0.1^d^	0.6±0.1^ab^	0.7±0.2^ab^	1.1±0.4^a^	0.5±0.2^bcd^	0.7±0.3^abcd^	0.8±0.2^abcd^	0.9±0.2^ab^
	VOC content [mg total LDM^-1^]^5^					15.6±1.2^b^				17.8±2.0^a^

RI = retention index; DAS = days after sowing; bold compounds represent the major volatile compounds of the cultivar; different letters within a row indicate significant differences at p ≤ 0.05. **^1^** Presented are mean RIs of samples under GC-FID conditions on an HP-5MS column relative to a series of n-alkanes. **^2^** Presented are mean percentages ± SD of volatile organic compounds of all analyzed basil leaf extracts (n = 8 leaf extracts per light treatment and DAS). **^3^** Percentage [%] ± SD represents total of listed volatile organic compounds. (Traces of eight identified compounds (namely β-pinene (RI 982), limonene (RI 1033), cis-linalool oxide (RI 1071), borneol (RI 1174), α-terpineol (RI 1196), chavicol (RI 1253), cis-methyl cinnamate (RI 1293), viridiflorol (RI 1596) and traces of two unidentified compounds (RI 1569, RI 1574) are excluded from the table.) **^4^** Presented are average VOC contents ± SD in percent [%] per gram of leaf dry matter (w/w) from four independent experimental replications per light treatment. **^5^** Presented are calculated average total VOC contents ± SD in mg total LDM^-1^ (leaf dry matter) across four spatial replications at harvest (35 DAS) per basil pot.

**Table 6:** Relative abundance of major volatile organic compounds identified in the leaves of *O. basilicum* L. cv. ‘Dark Opal’ as affected by LED light intensity treatments over time.

No.	Compound	RI^1^	I_Low_				I_High_			
			DAS 14	DAS 21	DAS 28	DAS 35	DAS 14	DAS 21	DAS 28	DAS 35
			Percent (%) of total volatile organic compound composition^2^							
1	*α*-pinene	938	0.4±0.0^cd^	0.4±0.0^cd^	0.4±0.0^cd^	0.4±0.1^bcd^	0.4±0.0^cd^	0.4±0.1^cd^	0.5±0.0^ab^	0.5±0.1^abc^
2	*β*-pinene	982	0.5±0.0^c^	0.6±0.1^bc^	0.6±0.1^bc^	0.7±0.1^b^	0.7±0.0^b^	0.6±0.1^bc^	0.9±0.1^a^	0.9±0.1^a^
3	*β*-myrcene	992	–	0.4±0.1^f^	0.4±0.1^def^	0.5±0.1^bcdef^	0.5±0.0^ef^	0.5±0.1^def^	0.6±0.0^ab^	0.7±0.1^a^
4	limonene	1033	0.7±0.1^abc^	0.6±0.1^abc^	0.6±0.1^bc^	0.6±0.1^abc^	0.7±0.0^a^	0.6±0.1^c^	0.7±0.1^ab^	0.6±0.1^abc^
5	**1,8-cineole**	**1036**	**4.4±0.3^d^**	**5.2±0.5^d^**	**5.6±0.7^d^**	**6.6±0.8^bcd^**	**6.0±0.0^abcd^**	**6.3±0.8^d^**	**8.2±1.0^abcd^**	**9.5±1.1^a^**
6	terpinolene	1095	3.3±0.7^abcd^	2.6±0.3^a^	2.3±0.2^abc^	2.2±0.1^abc^	2.9±0.3^a^	2.1±0.2^bc^	2.0±0.2^bc^	1.3±0.2^d^
7	**linalool**	**1101**	**1.6±0.7^e^**	**2.7±0.4^e^**	**4.5±0.8^d^**	**8.8±1.5^c^**	**5.4±0.4^d^**	**8.3±1.2^c^**	**16.0±2.3^b^**	**28.4±5.0^a^**
8	*α*-terpineol	1197	–	0.3±0.0^f^	0.4±0.0^e^	0.5±0.0^cd^	0.5±0.0^de^	0.6±0.1^c^	0.7±0.1^b^	1.0±0.1^a^
9	nerol	1227	0.9±0.1^b^	0.9±0.2^b^	0.9±0.1^b^	0.9±0.1^b^	0.9±0.1^b^	1.0±0.1^b^	1.5±0.2^a^	1.7±0.2^a^
10	**eugenol**	**1364**	**-**	**0.6±0.2^g^**	**1.0±0.3^fg^**	**3.2±0.8^cd^**	**2.1±0.9^defg^**	**4.9±1.0^bc^**	**4.6±0.3^b^**	**9.0±2.5^a^**
11	*β*-elemene	1403	–	0.4±0.1^d^	0.3±0.2^cd^	0.3±0.2^cd^	0.3±0.0^d^	0.5±0.1^c^	0.5±0.1^bcd^	0.8±0.1^a^
12	**methyl eugenol**	**1408**	**75.7±3.2^ab^**	**74.2±1.7^a^**	**72.3±2.2^a^**	**63.0±3.9^cd^**	**66.3±1.5^bc^**	**61.0±2.7^d^**	**48.4±3.8^f^**	**25.2±4.3^g^**
13	*α-trans*-bergamotene	1437	1.9±0.1^a^	1.7±0.1^bc^	1.7±0.1^bc^	1.7±0.1^abc^	1.5±0.1^c^	1.7±0.0^abc^	1.7±0.1^abc^	1.9±0.2^ab^
14	aromadendrene	1446	0.4±0.1^bcd^	0.3±0.0^f^	0.3±0.0^ef^	0.3±0.0^def^	0.5±0.1^ab^	0.4±0.1^bcd^	0.4±0.1^cde^	0.4±0.1^bcd^
15	*α*-humulene	1451	0.4±0.0^de^	0.3±0.0^ef^	0.3±0.0^ef^	0.4±0.0^cde^	0.2±0.0^f^	0.4±0.0^e^	0.5±0.1^bc^	0.8±0.1^a^
16	*β*-farnesene	1461	4.4±0.8^ab^	3.2±0.3^ab^	3.2±0.2^b^	3.4±0.3^ab^	4.0±0.5^ab^	3.7±0.2^a^	3.9±0.4^a^	3.3±0.3^ab^
17	*α*-amorphene	1471	0.8±0.1^a^	0.5±0.0^cd^	0.5±0.0^de^	0.5±0.0^e^	0.6±0.0^bcd^	0.5±0.0^bcde^	0.6±0.1^bcde^	0.6±0.0^bcd^
18	bicyclogermacrene	1498	1.8±0.2^f^	1.8±0.1^f^	2.0±0.1^cdef^	2.1±0.1^bc^	1.9±0.1^cdef^	2.1±0.2^bcd^	2.3±0.2^b^	3.1±0.2^a^
19	*γ*-cadinene	1513	0.5±0.0^e^	0.5±0.0^e^	0.7±0.1^d^	1.0±0.1^b^	0.8±0.1^cd^	0.9±0.1^bc^	1.2±0.2^abc^	1.3±0.2^a^
20	*δ*-cadinene	1521	–	0.1±0.1^f^	0.4±0.0^e^	0.6±0.0^bc^	0.3±0.0^f^	0.5±0.1^de^	0.8±0.1^b^	1.4±0.1^a^
21	*cis*-nerolidol	1529	0.6±0.0^fg^	0.6±0.1^fg^	0.6±0.1^efg^	0.7±0.0^de^	0.6±0.0^g^	0.7±0.0^de^	0.8±0.1^bcd^	1.2±0.1^a^
22	*α*-cadinol	1657	1.1±0.1^fg^	1.1±0.1^g^	1.2±0.2^efg^	1.1±0.0^g^	1.1±0.0^g^	1.3±0.1^def^	1.5±0.2^bcde^	2.3±0.1^a^
	TOTAL [%]^3^		99.4±0.4	99.4±0.2	99.4±0.1	99.1±0.3	98.8±0.3	98.3±0.4	98.8±0.4	98.3±0.3
	VOC content [%]^4^		0.9±0.0^bcd^	1.2±0.2^ab^	0.9±0.2^cd^	0.8±0.1^d^	1.5±0.3^a^	1.1±0.2^abc^	0.8±0.2^d^	0.8±0.2^d^
	VOC content [mg total LDM^-1^]^5^					2.6±0.2^b^				8.5±0.6^a^

RI = retention index; DAS = days after sowing; bold compounds represent the major volatile compounds of the cultivar; different letters within a row indicate significant differences at p ≤ 0.05. **^1^** Presented are mean RIs of samples under GC-FID conditions on an HP-5MS column relative to a series of n-alkanes. **^2^** Presented are mean percentages ± SD of volatile organic compounds of all analyzed basil leaf extracts (n = 8 leaf extracts per light treatment and DAS). **^3^** Percentage [%] ± SD represents total of listed volatile organic compounds. (Traces of ten identified compounds (namely sabinene (RI 978), cis-linalool oxide (RI 1071), borneol (RI 1174), methyl chavicol (RI 1203), chavicol (RI 1256), β-cubebene (RI 1384), trans-methyl cinnamate (RI 1390), germacrene D (RI 1479), viridiflorol (RI 1596), epi-α-cadinol (RI 1633) and traces of three unidentified compounds (RI 1015, RI 1120, RI 1548) are excluded from the table.) **^4^** Presented are average VOC contents ± SD in percent [%] per gram of leaf dry matter (w/w) from four independent experimental replications per light treatment. **^5^** Presented are calculated average total VOC contents ± SD in mg total LDM^-1^ (leaf dry matter) across four spatial replications at harvest (35 DAS) per basil pot.

The complexity of VOC profiles increased with the basils’ progressing development. In example, while leaf extracts of 14-day-old cv. Anise seedlings were characterized by nine and twelve major volatiles under I_Low_ and I_High_, respectively, the total of detected VOCs increased weekly and reached their highest total in 35-day-old basil plants (**Table 3**).

Regardless of the light intensities applied, each cultivar underwent analogous compositional changes throughout the trial period. In example, under both light intensity treatments, percentages of linalool and *trans*-methyl cinnamate increased and percentages of methyl chavicol and methyl eugenol decreased in the developing ‘Cinnamon’ cultivar (**Table 5**).

However, in comparison to the compositional changes observed in the cultivars under I_High_, the same directional changes occurred time-delayed under I_Low_. In example, while the percentage of methyl chavicol reduced from ~ 17.7 % at 14 DAS to ~ 12.0 % at 21 DAS and ~ 9.1 % at 28 DAS under I_High_ in the basil cultivar ‘Cinnamon’, a comparable drop was observed one week later under I_Low_ as the percentage of methyl chavicol decreased from ~ 20.5 % at 21 DAS to ~ 15.3 % at 28 DAS and finally ~ 8.7 % at 35 DAS (**Table 5**). In the same manner, the percentage of linalool increased from ~ 5.4 % at 14 DAS to ~ 8.3 % at 21 DAS under I_High_, while linalool increased in the same way (from ~ 4.5 to ~ 8.8 %) two weeks later between 28 DAS and 35 DAS under I_Low_ in the cultivar ‘Dark Opal’, (**Table 6**).

So far, not many studies have investigated compositional differences in *Ocimum basilicum* L. under different (LED) light intensities, but their outcomes are highly consistent with our findings. In example, Chang et al. (2008) and Walters et al. (2021) reported increasing linalool concentrations in the green basil cultivars ‘Genovese’ and ‘Nufar’, respectively, with increasing light intensities, which agrees with our findings in the linalool-containing cultivars ‘Cinnamon’, ‘Dark Opal’ and ‘Thai Magic’. Also, the observed relative decrease of methyl eugenol with increasing light intensities in cv. Genovese (Chang et al., 2008) was observed in the three cultivars that were characterized by elevated concentrations of methyl eugenol (cv. Cinnamon, cv. Dark Opal and cv. Thai Magic). The authors also described fairly constant as well as slightly increasing relative concentrations of 1,8-cineole in cv. Genovese and cv. Nufar, respectively. This is also congruent with the indifferent relative concentrations of 1,8-cineole in the cultivars ‘Anise’, ‘Cinnamon’ and ‘Thai Magic, and the slight upwards trend of 1,8-cineole concentrations detected in cv. Dark Opal with increasing light intensity. In addition, the fluctuating relative eugenol concentrations reported by Chang et al. (2008) and Walters et al. (2021) were also detected in our study in the eugenol-containing cultivars ‘Cinnamon’ and ‘Dark Opal’. Alike the findings observed by Walters et al. (2021) in 14-day-old cv. Nufar seedlings, we observed no differences in relative methyl chavicol concentrations between both light treatments throughout the trial period in cv. Cinnamon and cv. Dark Opal and 14 DAS in cv. Anise. In contrast, decreased relative methyl chavicol concentrations under I_High_ were observed in older cv. Anise at 21, 28 and 35 DAS.

During the last few years, great progress has been made in the elucidation of most of the genes, transcription factors, enzymes, and substrates involved in the biosynthesis of the individual terpenes and phenylpropenes detected in our study. This led to coherent proposals of their biosynthetic pathways in the peltate glands of basil (Gang et al., 2001; Iijima et al., 2004; Kapteyn et al., 2007; Zvi et al., 2012; Dhar et al., 2020). *In vivo* however, the individual VOC profiles depend greatly on enzyme abundance and activity, their substrate specificity and availability (Lewinsohn et al., 2000; Muñoz-Bertomeu et al., 2006; Xie et al., 2008; Singh et al., 2015; Yahaa et al., 2019), all of which can be highly affected by abiotic factors (including drought stress (Mandoulakani et al., 2017), carbon dioxide concentration and temperature (Chang et al., 2005; Tursun & Telci, 2020), nutrients and biofertilizers (Hanif et al., 2017, Dehsheikh et al., 2020), light quality (Carvalho et al., 2016; Hosseini et al., 2018; Milenković et al., 2019; Tabbert et al., 2022) and elicitors (Deschamps & Simon, 2006; Loni et al., 2021)). The dynamics are further complicated as basils’ VOC compositions do not only depend on individual leaf maturation (Lewinsohn et al., 2000) but also considerably on the position of the basil leaf along the stem (Fischer et al., 2011). Thus, the underlying processes that regulate these compositional changes are exceptionally complex.

Nevertheless, the weekly assessments of the basils’ VOC compositions clearly show that each basil cultivar undergoes fundamentally well-coordinated compositional changes. As the detected compositional changes were identical under both light intensity treatments and occurred only time-delayed under I_Low_ in comparison to I_High_, it becomes evident that the observed compositional differences in our study (as well as the differences observed by Chang et al., 2008 and Walters et al., 2021) are related to the cultivars’ developmental stage and thus, growth rates. The high correlations between the basils’ complex VOC profiles and their morphological stages as determined by principal component regressions (**Table 7**) strongly support this developmental stage-dependency.

**Table 7:** Correlation between basils’ complex VOC profiles and their morphological stage.

Dependent variable	Goodness of fit^1^			
	‘Anise’	‘Cinnamon’	‘Dark Opal’	‘Thai Magic’
Plant height	0.88	0.87	0.85	0.82
Number of leaf pairs	0.75	0.96	0.78	0.87
Number of branch pairs	0.76	0.90	0.90	0.92

^1^ Summarized are the coefficients of determination (R^2^) that describe the goodness of fit of the selected dependent variable on the basils’ aroma profiles as determined by principal component regressions (PCRs).

Different VOC accumulation patterns were observed between the investigated basil cultivars (**Figures 5Q–S**). While VOC concentrations per gram of leaf dry matter (L_DM_) gradually increased in the cultivars ‘Anise’, ‘Cinnamon’ and ‘Thai Magic’ under both light intensity treatments during the trial period, a decreasing trend in VOC concentrations was observed in cv. Dark Opal. That VOC accumulation patterns strongly deviate within the species *Ocimum basilicum* has already been demonstrated by Macchia et al., 2006 and Szabó & Bernáth, 2002.

Within each cultivar, VOC concentrations generally did not differ between I_Low_ and I_High_ during each investigated time point, however, exceptions were observed in the cultivars ‘Dark Opal’ and ‘Thai Magic’ (**Figures 5Q–S**). The reason for the indifferent VOC concentrations observed under both light intensity treatments at each time point are likely to be attributed to the fact that the examined leaf extracts contained all developed leaves per basil plant irrespective of leaf age and maturation: Generally, glandular trichome densities decline while leaves age and expand. Though young leaves generally contain lower VOC quantities than older leaves, younger leaves tend to have higher VOC concentrations due to their lower weight (Fischer et al., 2011). The significantly lower VOC concentration detected under I_Low_ in comparison to I_High_ at 14 DAS in ‘Dark Opal’ (**Figure 5S**) may be ascribed to the cultivars’ limited photosynthetic capabilities under low light conditions as discussed before in section 3.4 and hence, a presumably lower availability of photosynthates for secondary metabolism. The slightly increased VOC concentration observed under I_Low_ in comparison to I_High_ at 35 DAS in cv. Thai Magic (**Figure 5T**) may indicate that this cultivar reaches its maximum VOC accumulation before flower buds arise (**Figure 4D**) after which VOC concentrations decrease – an accumulation pattern that has previously been described for the *Ocimum basilicum* cultivars ‘Rit-Sat’ and ‘Lengyel’ (Szabó & Bernáth, 2002) as well as for *Ocimum ciliatum* (Moghaddam et al., 2015). However, further experiments are needed to prove these hypotheses.

When comparing total VOC yields produced at the end of the experiment (35 DAS), all cultivars had accumulated substantially greater VOC quantities under I_High_ than basils of the same age under I_Low_ due to the cultivars’ overall greater leaf biomasses accumulated under I_High_ (**Table 2**, **Figures 5U–X**). In comparison to VOC yields under I_Low_, yields were elevated by ~ 12.4, ~ 45.6, ~ 60.8 and ~ 69.2 % under I_High_ in cultivars ‘Cinnamon’, ‘Thai Magic’, ‘Anise’ and ‘Dark Opal’, respectively.

It is well known that the flavor of basil is due to the presence and individual concentration of specific terpenes and phenylpropenes (for basil aroma descriptions see Patel et al., 2021). As the leaf extracts of the cultivars ‘Anise’ and ‘Thai Magic’ contained high concentrations of methyl chavicol throughout the investigation, both cultivars should be characterized by strong anise-like/licorice flavors regardless of their developmental stage and light intensity treatment applied (**Tables 3**, **4**). With relative concentrations of 1,8-cineole between 6.8 and 9.3 %, ‘Anise’ should also include a noticeable eucalyptus aroma (**Table 3**). However, as shown by Lee et al. (2017), a trained panel was able to clearly discriminate between two basil cultivars that contained the same array of flavoring volatiles in different relative ratios. That indicates that the specific flavors of the cultivars ‘Cinnamon’ and ‘Dark Opal’ may tremendously change over time: While relatively high concentrations of methyl chavicol (imparting an anise-like/licorice aroma) and methyl eugenol (imparting a weak clove-like aroma) were detected during ‘Cinnamons’ early development, more mature ‘Cinnamon’ basils were characterized by relatively high concentrations of linalool (imparting a sweet floral aroma) and *trans*-methyl cinnamate (imparting a cinnamon-like aroma) (**Table 5**). Similarly, while methyl eugenol (weak clove-like aroma) clearly dominated in young ‘Dark Opal’, the relative abundances of linalool (sweet floral aroma), eugenol (clove-like aroma) and 1,8-cineole (eucalyptus aroma) increased greatly over time (**Table 6**).

Though concrete health risks for humans have not been confirmed, methyl chavicol (estragole) and methyl eugenol have shown to induce dose-dependent genotoxic and carcinogenic effects in multiple independent animal trails (NTP, 2000; Martins et al., 2012) which raised toxicological concerns of these naturally occurring substances. On grounds of the preventive protection of public health, legislators banned the addition of methyl chavicol and methyl eugenol as food additives and set maximum levels of these substances if naturally present in flavorings and food ingredients (Regulation No 1334, 2008). Consequently, basil producers and food processing industries are obligated to exploit possibilities to largely lower these two critical constituents.

As the main VOC in cv. Anise and cv. Thai Magic, methyl chavicol was found in consistently high percentages regardless of DAS and light intensity treatment (Tab. 3, 4). However, independent of the light intensity treatment, the percentages of methyl chavicol and methyl eugenol continuously decreased during ontogenesis of cv. Cinnamon (Tab. 5). Thus, by thoughtfully managing the time of harvest, levels of both critical constituents can be significantly reduced. Further, with 75.5 % under I_Low_ and 66.3 % under I_High_ at DAS 14, the percentages of methyl eugenol are indifferently high under both light treatments in cv. Dark Opal (Tab. 6). While the percent share reduces only to 63 % under I_Low_, the percent share significantly drops to 25.2 % under I_High_ by the end of the experiment. Hence, cultivating cv. Dark Opal under low light intensities is not only unsuitable in terms of low yield and insufficient morphological development, but also with regard to methyl eugenol as a compound of toxicological concern.

In addition, as observed in cv. Dark Opal, *via* suitable light intensity settings, contents of (critical) compounds can be controlled and significantly reduced.

### 3.7 In contrast to purple basil, green basil cultivars use energy more efficiently under I_Low_

Despite the accelerated basil development observed under I_High_, the green-leafed cultivars ‘Anise’, ‘Cinnamon’ and ‘Thai Magic’ converted the available light energy more effectively into biomass under I_Low_ than the basil plants that were cultivated under I_High_ (**Table 8**). If these three cultivars were to be grown until marketability (based on the morphological criteria described in section 3.2), more than 50 % of electrical energy could be saved under I_Low_ in comparison to I_High_, while cultivar-dependent yields would only be decreased by approximately 3-11 % (**Table 9**). In theory, a little more than one additional crop cycle per year could be generated under I_High_. In contrast, year-round basil cultivations under I_Low_ would save ~ 57 % in energy usage while cultivar-dependent productivity losses would be between 15-19 % (**Table 10**). Hence, the great low light adaptability of the green basil cultivars (as described in section 3.4) ultimately resulted in improved biomass efficiencies under I_Low_ when compared to the biomass efficiencies reached under I_High_.

**Table 8:** Biomass efficacy of four *Ocimum basilicum* L. cultivars grown under two different light intensity treatments.

Basil cultivar	Biomass efficiency [g kWh^-1^]^1^		
	I_Low_	I_High_	*P*
‘Anise’	5.6 ± 0.1^a^	3.4 ± 0.6^b^	0.03
‘Cinnamon’	10.0 ± 1.3^a^	5.2 ± 0.8^b^	0.01
‘Dark Opal’	2.9 ± 0.1*^ns^*	3.4 ± 0.6*^ns^*	*ns*
‘Thai Magic’	6.7 ± 0.6^a^	3.9 ± 0.2^b^	0.01

Different letters within a row indicate significant differences at p ≤ 0.05; P = significance level; ns = not significant.**^1^** Presented are calculated average basil fresh weights ± SD (n = 4) produced per kilowatt hour consumed under the experimental area of 1.2 m^2^ by the end of the trial period (35 days after sowing) from four independent spatial replications per light treatment.

**Table 9:** Energy consumption and biomass at marketability of four *Ocimum basilicum* L. cultivars grown under two different light intensities.

Basil cultivar	Marketability^1^ [DAS]		Energy consumption [kWh] at marketability		Energy savings [%]	FW [g pot^-1^] at marketability^2^		Productivity loss [%]
	I_High_	I_Low_	I_High_	I_Low_		I_High_	I_Low_	
‘Anise’	29	33	327.9	161.1	50.9	~ 11.9	~ 11.5	~ 9.1
‘Cinnamon’	27	30	305.3	146.4	52.4	~ 18.7	~ 17.0	~ 3.4
‘Dark Opal’	32	*na*	361.8	*na*	*na*	~ 14.9	*na*	*na*
‘Thai Magic’	30	33	339.2	161.1	52.5	~ 15.0	~ 13.4	~ 10.7

DAS = days after sowing; FW = fresh weight; I_High_ = High light intensity; I_Low_ = Low light intensity; na = not available. **^1^** Marketability is defined as plant height ≥ 15 cm and/or number of leaf pairs ≥ 4 [by our cooperation partners Oderbruch Müller, an organic nursery in Bad Freienwalde, Germany]; see also **Figures 5A–H**. **^2^** Basil fresh weights [g pot^-1^] at marketability were calculated using each cultivars’ polynomial biomass rates under each light treatment (R^2^ > 0.99).

**Table 10:** Achievable crop cycles, biomass production, and its energy consumption per year when grown until marketability.

Basil cultivar	Crop cycles per year^1^		FW[kg area^-1^ y^-1^]^2^		Productivity loss [%]	Energy consumption [kWh y^-1^]		Energy savings [%]
	I_High_	I_Low_	I_High_	I_Low_		I_High_	I_Low_	
‘Anise’	12.6	11.1	~ 10.8	~ 9.2	~ 14.8	4126.6	1781.7	56.8
‘Cinnamon’	13.5	12.2	~ 18.2	~ 14.9	~ 18.1			
‘Dark Opal’	11.4	*na*	~ 12.2	*na*	*na*			
‘Thai Magic’	12.2	11.1	~ 13.2	~ 10.7	~ 18.9			

FW = Fresh weight; na = not applicable. ^1^ From seed to marketability (see also **Table 9**). ^2^ Fresh weight [kg] per experimental area (1.2 m^2^) and year were calculated by multiplying the calculated average fresh weights at marketability with the number of possible plant pots (n = 72) and the number of possible crop cycles per year.

In contrast, biomass efficiencies of cv. Dark Opal were statistically indifferent between the two light intensity treatments (**Table 8**). Because cv. Dark Opal did not reach the marketability threshold under I_Low_ during the trial period, a cultivation under I_Low_ light conditions would result in the lowest number of crop cycles per year and the greatest productivity losses of all investigated cultivars (**Tables 9**, **10**).

### 3.8 Improvement of basil production *via* eustress management

In general, beneficial effects induced by small doses and/or durations of stress factors are called eustressor effects (Vázquez-Hernández et al., 2019), and basil producers may be encouraged to provoke slight light and/or shade stresses in basil to stimulate different beneficial effects on their performance and/or human health: Even though the observed accumulations of anthocyanins in the top leaves of cv. Anise, cv. Cinnamon and cv. Thai Magic under I_High_ represent direct stress responses triggered by the intense light conditions they grew into to protect their chloroplasts from photoinhibitory and photooxidative effects (as described in section 3.3), other diverse protective roles are ascribed to anthocyanins as members of the flavonoid group (Lila, 2004). These phytochemicals are known to enhance the plants’ resistance to the effects of chilling and freezing and increase their resistance to herbivores and pathogens (Gould, 2004). In addition, numerous protective roles to human health (from antioxidative and free-radical scavenging capabilities, obesity prevention, amelioration of hyperglycemia, neuroprotective effects to positive influences on vascular functions) have been attributed to anthocyanins (well summarized by Lila, 2004). In addition, the light stress under I_High_ resulted in accelerated basil growths, accompanied with enhanced VOC maturations and high yields (as described in sections 3.1, 3.2 and 3.6). On the other hand, the shade stress under I_Low_ resulted in consumer-preferred leaf appearances and producer-preferred biomass efficiencies (as described in sections 3.4 and 3.7). Hence, basil producers may be interested in carefully managing light and/or shade stresses to stimulate specific positive effects of interest in basil and/or to improve their production efficiencies (Vázquez-Hernández et al., 2019).

### 3.9 Conclusion

From seed to marketability, the broad-bandwidth LED light spectrum including FR light with elevated R and B light fractions enabled an adequate growth and development of all four investigated basil cultivars (‘Anise’, ‘Cinnamon’, ‘Dark Opal’ and ‘Thai Magic’) under both rising light intensity conditions applied in this study.

In comparison to the I_Low_ light conditions, I_High_ resulted in an accelerated development and thus expedited marketability of all investigated basil cultivars. However, exposure to light intensities above 300 µmol m^-2^ s^-1^ caused adverse quality-reducing effects in the green-leafed cultivars ‘Anise’, ‘Cinnamon’ and ‘Thai Magic’ and should therefore not be exceeded.

In contrast, the applied I_Low_ light conditions resulted in consumer-preferred appearances and greater biomass efficiencies and appear to be the result of a great low light adaptability of green-leafed basil cultivars. Despite the time-delayed marketability (based on morphological criteria) under I_Low_ in comparison to I_High_, the superior visual quality of the green-leafed cultivars in combination with the significantly reduced energy consumptions under I_Low_ can ultimately result in greater revenues for basil producers.

Though purple-leafed cultivar ‘Dark Opal’ was able to mitigate high intensity light stress responses under I_High_ in comparison to the green-leafed cultivars, its indoor production until the common marketability criteria (plant height ≥ 15 cm and/or leaf pairs ≥ 4) are reached remains the least economical due to the high energy consumptions necessary, the comparatively low yields and number of annual crop cycles possible.

However, basil cultivar-specific VOC contents and profiles proved to change tremendously over time in a developmental stage-correlated manner. Thus, consumer flavor preferences of differently aged basil plants should be further explored, and time of harvest should be individually reconsidered [especially as the demand and popularity of microgreens is on the rise (Kyriacou et al., 2016; Mir et al., 2016)].

## Data availability statement

The original contributions presented in the study are included in the article. Further inquiries can be directed to the corresponding authors.

## Author contributions

JT: conceptualization, project administration, formal analysis, validation, investigation, data curation, and writing – original draft preparation. DR: methodology, formal analysis, validation. AK: conceptualization, and writing – review and editing. HS: conceptualization, project administration, methodology, funding acquisition, resources, and supervision. All authors contributed to the article and approved the submitted version.

## Funding

This work was supported and funded by the European Innovation Partnership for Improvement of Agricultural Productivity and Sustainability (grant number 204016000016/80168353) *via* the European Agricultural Fund for Rural Development.

## Acknowledgments

We sincerely thank Maik Repnack and his team for technical support, Roland Buchhorn, Claudia Könecke and Heike Bäumer for cultivation assistance and Dominique Conrad and Mario Harke for assisting in data curation. We further thank Matthias Melzig from Free University Berlin for his support.

## Conflict of interest

HS is the owner of the company Consulting & Project Management for Medicinal and Aromatic Plants.

The remaining authors declare that the research was conducted in the absence of any commercial or financial relationship that could be construed as a potential conflict of interest.

## Publisher’s note

All claims expressed in this article are solely those of the authors and do not necessarily represent those of their affiliated organizations, or those of the publisher, the editors and the reviewers. Any product that may be evaluated in this article, or claim that may be made by its manufacturer, is not guaranteed or endorsed by the publisher.
