# Corrigendum: Facing energy limitations – approaches to increase basil (*Ocimum basilicum* L.) growth and quality by different increasing light intensities emitted by a broadband LED light spectrum (400-780 nm)

**DOI:** 10.3389/fpls.2023.1200910

**Published:** 2023-07-04

**Authors:** Jenny Manuela Tabbert, David Riewe, Hartwig Schulz, Andrea Krähmer

**Affiliations:** ^1^ Julius Kühn Institute – Federal Research Centre for Cultivated Plants, Institute for Ecological Chemistry, Plant Analysis and Stored Product Protection, Berlin, Germany; ^2^ Institute of Pharmacy, Freie Universität Berlin, Berlin, Germany; ^3^ Consulting & Project Management for Medicinal and Aromatic Plants, Stahnsdorf, Germany

**Keywords:** light-emitting diode (LED), morphology, essential oil, volatile organic compound (VOC), energy use efficiency (EUE)

In the published article, there was an error in the author list, and David Riewe was erroneously excluded.

The corrected author list appears below.

Jenny Manuela Tabbert^1,2*^, David Riewe^1^, Hartwig Schulz^1,3†^ and Andrea Krähmer^1*^

A correction has been made to the **Citation** statement. The citation previously stated:

“Tabbert JM, Schulz H and Krähmer A (2022) Facing energy limitations – approaches to increase basil (*Ocimum basilicum* L.) growth and quality by different increasing light intensities emitted by a broadband LED light spectrum (400-780 nm). *Front. Plant Sci*. 13:1055352. doi: 10.3389/fpls.2022.1055352”

The corrected citation appears below:

“Tabbert JM, Riewe D, Schulz H and Krähmer A (2022) Facing energy limitations – approaches to increase basil (*Ocimum basilicum* L.) growth and quality by different increasing light intensities emitted by a broadband LED light spectrum (400-780 nm). *Front. Plant Sci*. 13:1055352. doi: 10.3389/fpls.2022.1055352”

A correction has been made to the **Copyright** statement.

The copyright previously stated:

“^©^ 2022 Tabbert, Schulz and Krähmer. This is an open-access article distributed under the terms of the Creative Commons Attribution License (CC BY). The use, distribution or reproduction in other forums is permitted, provided the original author(s) and the copyright owner(s) are credited and that the original publication in this journal is cited, in accordance with accepted academic practice. No use, distribution or reproduction is permitted which does not comply with these terms.”

The corrected copyright appears below:

“^©^ 2022 Tabbert, Riewe, Schulz and Krähmer. This is an open-access article distributed under the terms of the Creative Commons Attribution License (CC BY). The use, distribution or reproduction in other forums is permitted, provided the original author(s) and the copyright owner(s) are credited and that the original publication in this journal is cited, in accordance with accepted academic practice. No use, distribution or reproduction is permitted which does not comply with these terms.”

A correction has been made to the **Author Contribution** statement. This paragraph previously stated:

“JT: conceptualization, project administration, formal analysis, validation, investigation, data curation, and writing – original draft preparation. AK: conceptualization, and writing – review and editing. HS: conceptualization, project administration, methodology, funding acquisition, resources, and supervision. All authors contributed to the article and approved the submitted version.”

The corrected paragraph appears below.

“JT: conceptualization, project administration, formal analysis, validation, investigation, data curation, and writing – original draft preparation. DR: methodology, formal analysis, validation. AK: conceptualization, and writing – review and editing. HS: conceptualization, project administration, methodology, funding acquisition, resources, and supervision. All authors contributed to the article and approved the submitted version.”

In the published article, there was an error in **Table 4**: **Relative abundance of major volatile organic compounds identified in the leaves of *O. basilicum* L. cv. ‘Dark Opal’ as affected by LED light intensity treatments over time**. **Table 4** erroneously shows the compounds of ‘Dark Opal’ but has to show the compounds of cv. ‘Thai Magic’ to match the references in the text and to stay in chronological order.

**Table 4:** Relative abundance of major volatile organic compounds identified in the leaves of *O. basilicum* L. cv. ‘Thai Magic’ as affected by LED light intensity treatments over time.

No.	Compound	RI^1^	I_Low_				I_High_				
			DAS 14	DAS 21	DAS 28	DAS 35	DAS 14		DAS 21	DAS 28	DAS 35
			Percent (%) of total volatile organic compound composition^2^								
1	*α*-pinene	938	–	–	0.3±0.0	0.2±0.0	–		–	0.2±0.0	0.2±0.0
2	*β*-pinene	982	–	0.4±0.0	0.3±0.0	0.3±0.0	–		0.4±0.0	0.4±0.1	0.4±0.0
3	*β*-myrcene	992	–	–	0.3±0.0	0.3±0.0	–		–	0.4±0.1	0.3±0.0
4	limonene	1033	0.7±0.2^abc^	0.7±0.1^a^	0.6±0.0^ab^	0.5±0.1^bc^	0.7±0.0^a^		0.7±0.1^ab^	0.6±0.1^abc^	0.5±0.0^c^
5	1,8-cineole	1036	2.5±0.5^d^	3.4±0.4^c^	3.4±0.4^c^	3.6±0.3^bc^	3.9±0.5^abc^		4.4±0.5^a^	4.2±0.6^ab^	3.9±0.4^abc^
6	*trans*-*ß*-ocimene	1050	0.7±0.1^e^	0.8±0.1^de^	1.1±0.1^c^	1.4±0.2^b^	1.0±0.1^c^		0.9±0.1^cd^	1.7±0.3^ab^	1.8±0.2^a^
7	terpinolene	1095	2.9±0.9^a^	2.4±0.6^a^	1.8±0.2^b^	1.2±0.3^c^	3.1±0.8^a^		2.2±0.6^a^	1.3±0.3^c^	0.8±0.1^c^
8	linalool	1100	–	0.4±0.1^c^	0.6±0.1^bc^	0.9±0.3^ab^	–		1.2±0.8^abc^	1.3±0.4^a^	1.2±0.3^a^
9	camphor	1154	1.1±0.1^c^	1.3±0.3^bc^	1.3±0.1^bc^	1.5±0.2^b^	1.7±0.4^ab^		2.0±0.3^a^	1.9±0.2^a^	1.4±0.2^bc^
10	borneol	1174	–	0.4±0.1	0.5±0.1	0.7±0.1	–		0.5±0.1	0.8±0.2	1.4±0.3
11	*α*-terpineol	1197	0.4±0.0^c^	0.4±0.0^c^	0.5±0.1^bc^	0.5±0.0^b^	0.5±0.1^bc^		0.5±0.0^b^	0.7±0.1^a^	0.7±0.1^a^
**12**	**methyl chavicol**	**1204**	**69.7±1.9^abcde^**	**73.0±2.6^abc^**	**73.5±2.0^a^**	**71.7±1.1^abcd^**	**68.7±4.2^cde^**		**68.6±1.8^de^**	**66.4±3.3^e^**	**70.1±2.3^abcde^**
13	*trans*-methyl cinnamate	1390	1.1±0.1^a^	0.7±0.1^b^	0.6±0.1^c^	0.3±0.0^de^	0.8±0.1^b^		0.4±0.1^cd^	0.2±0.0^e^	–
14	*β*-elemene	1403	–	–	0.3±0.0^b^	0.4±0.1^b^	–		–	0.4±0.1^b^	0.7±0.1^a^
15	methyl eugenol	1406	7.2±1.8^a^	3.0±0.4^bc^	2.2±0.6^cd^	1.4±0.2^d^	4.2±0.6^ab^		2.0±0.4^d^	1.1±0.1^e^	0.9±0.2^e^
16	*α-trans*-bergamotene	1437									
17	aromadendrene	1446	4.6±0.1^b^	4.4±0.8^b^	4.3±0.3^b^	5.2±0.8^ab^	5.4±0.5^ab^		5.7±0.8^ab^	6.5±1.1^a^	5.3±1.1^ab^
18	*β*-farnesene	1460	3.8±0.7^a^	2.6±0.3^b^	2.6±0.4^b^	2.2±0.3^bc^	3.5±0.6^a^		2.1±0.3^bc^	2.0±0.5^bc^	1.6±0.4^c^
19	bicyclogermacrene	1497	1.0±0.2^de^	0.8±0.0^e^	1.1±0.2^bcd^	1.4±0.1^ab^	1.2±0.2^bcde^		1.1±0.2^cde^	1.6±0.4^abc^	1.8±0.3^a^
20	*γ*-cadinene	1514	–	–	0.3±0.1^c^	0.4±0.1^bc^	–		–	0.5±0.1^ab^	0.5±0.1^a^
21	*δ*-cadinene	1521	–	–	0.3±0.1^c^	0.5±0.2^bc^	–		–	0.7±0.1^ab^	0.9±0.2^a^
22	*cis*-nerolidol	1529	–	0.4±0.0^d^	0.6±0.1^c^	0.8±0.1^b^	–		0.8±0.2^bc^	1.1±0.2^ab^	1.3±0.2^a^
23	spathulenol	1578	0.5±0.1^c^	1.2±0.5^ab^	0.7±0.4^bc^	0.6±0.2^bc^	1.0±0.2^b^		2.0±0.5^a^	0.6±0.2^bc^	0.6±0.1^bc^
24	*epi*-*α*-cadinol	1633	–	–	–	0.3±0.0^b^	–		–	0.3±0.0^a^	0.4±0.1^a^
25	*α*-cadinol	1657	0.4±0.1^f^	0.7±0.1^e^	1.1±0.2^cd^	1.5±0.2^bc^	0.8±0.2^def^		1.3±0.3^bcd^	1.9±0.4^ab^	2.3±0.3^a^
	TOTAL [%]^3^		96.7±0.7	97.5±0.6	97.9±0.4	97.6±0.4	97.2±0.6		96.6±0.2	96.5±1.1	97.6±0.9
	VOC content [%]^4^		0.9±0.1^bc^	0.9±0.2^bc^	1.2±0.2^abc^	1.3±0.2^ab^	0.8±0.2^c^		0.7±0.2^c^	1.4±0.4^a^	0.9±0.3^bc^
	VOC content [mg total LDM^-1^]^5^					10.5±0.5^b^					19.2±0.5^a^

RI, retention index; DAS, days after sowing; the bold compound represents the major volatile compound of the cultivar; different letters within a row indicate significant differences at p ≤ 0.05. **^1^** Presented are mean RIs of samples under GC-FID conditions on an HP-5MS column relative to a series of n-alkanes. **^2^** Presented are mean percentages ± SD of volatile organic compounds of all analyzed basil leaf extracts (n = 8 leaf extracts per light treatment and DAS). **^3^** Percentage [%] ± SD represents total of listed volatile organic compounds. (Traces of nine identified compounds (namely camphene (RI 954), sabinene (RI 978), cis-sabinene hydrate (RI 1072), chavicol (RI 1253), nerol (RI 1227), cis-methyl cinnamate (RI 1294), eugenol (RI 1364), α-humulene (RI 1452), α-amorphene (RI 1471), germacrene D (RI 1479), trans-cadina-1,4-diene (RI 1534), viridiflorol (RI 1596) and traces of four unidentified compounds (RI 1015, RI 1120, RI 1261, RI 1557) are excluded from the table.) **^4^** Presented are average VOC contents ± SD in percent [%] per gram of leaf dry matter (w/w) from four independent experimental replications per light treatment. **^5^** Presented are calculated average total VOC contents ± SD in mg total LDM^-1^ (leaf dry matter) across four spatial replications at harvest (35 DAS) per basil pot.

The corrected **Table 4** Relative abundance of major volatile organic compounds identified in the leaves of *O. basilicum* L. cv. ‘Thai Magic’ as affected by LED light intensity treatments over time and its caption appear below.

In the published article, there was an error in **Table 5** **Relative abundance of major volatile organic compounds identified in the leaves of *O. basilicum* L. cv. ‘Thai Magic’ as affected by LED light intensity treatments over time**. **Table 5** erroneously shows the compounds of ‘Thai Magic’ but has to show the compounds of cv. ‘Cinnamon’ to match the references in the text and to stay in chronological order.

**Table 5:** Relative abundance of major volatile organic compounds identified in the leaves of *O. basilicum* L. cv. ‘Cinnamon’ as affected by LED light intensity treatments over time.

No.		Compound	RI^1^		I_Low_					I_High_			
					DAS 14	DAS 21	DAS 28	DAS 35		DAS 14	DAS 21	DAS 28	DAS 35
					Percent (%) of total volatile organic compound composition^2^								
1		1,8-cineole	1036		3.0±0.7^ab^	2.5±0.6^ab^	2.4±0.8^b^	3.4±0.7^a^		3.3±0.7^ab^	3.1±0.5^ab^	2.7±0.4^ab^	2.6±0.6^ab^
2		*trans*-*ß*-ocimene	1049		0.6±0.0	0.6±0.1	0.5±0.2	0.5±0.1		0.6±0.3	0.7±0.1	0.5±0.0	0.6±0.3
3		terpinolene	1094		1.6±0.4^a^	0.9±0.3^bc^	0.6±0.2^bc^	0.4±0.1^c^		1.6±0.4^a^	1.0±0.4^b^	0.5±0.1^bc^	0.5±0.1^bc^
**4**		**linalool**	**1101**		**7.9±2.2^e^**	**13.8±2.5^d^**	**18.8±1.8^c^**	**23.7±3.1^ab^**		**13.6±3.0^d^**	**20.1±2.8^bc^**	**27.2±3.1^a^**	**23.5±5.0^abc^**
5		camphor	1153		0.8±0.1	0.6±0.1	0.6±0.2	0.5±0.2		0.8±0.1	0.8±0.3	0.5±0.2	0.5±0.2
6		*α*-terpineol	1184		0.9±0.1	0.8±0.2	0.9±0.3	0.7±0.2		0.7±0.1	0.9±0.2	0.7±0.3	0.7±0.5
**7**		**methyl chavicol**	**1203**		**26.8±11.3^a^**	**20.5±5.4^ab^**	**15.3±7.1^bc^**	**8.7±5.0^c^**		**17.7±10.0^abc^**	**12.0±4.8^bc^**	**9.1±6.5^bc^**	**9.8±4.2^bc^**
9		eugenol	1364		1.7±1.1	1.6±0.5	3.1±1.6	5.2±4.8		3.8±2.2	1.5±0.7	5.9±5.1	2.1±2.5
10		***trans*-methyl cinnamate**	**1391**		**8.7±3.8^d^**	**30.2±6.0^abc^**	**35.1±8.2^a^**	**33.3±8.4^a^**		**20.2±6.4^bcd^**	**34.4±6.2^a^**	**27.5±5.6^ab^**	**36.9±12.1^a^**
**11**		*β*-elemene	1403		0.7±0.0	0.5±0.1	0.6±0.2	0.9±0.3		0.4±0.0	0.6±0.2	0.7±0.2	0.9±0.3
12		**methyl eugenol**	**1406**		**27.5±10.8^a^**	**13.2±3.7^abc^**	**6.7±1.9^def^**	**4.0±2.9^efg^**		**18.2±8.9^abcd^**	**7.5±1.6^cde^**	**3.3±2.2^fg^**	**0.9±0.2^g^**
13		aromadendrene	1446		2.2±0.6^abc^	1.4±0.5^abcd^	1.3±0.2^d^	1.3±0.4^cd^		2.3±0.6^ab^	1.9±1.1^bcd^	2.0±1.2^bcd^	0.9±0.3^d^
14		*α*-humulene	1451		–	–	0.4±0.1^b^	0.6±0.2^ab^		–	0.4±0.0^b^	0.8±0.3^a^	0.7±0.2^ab^
15		*β*-farnesene	1460		5.6±0.7^b^	4.3±0.5^c^	3.0±0.5^de^	2.2±0.5^ef^		6.7±1.0^a^	3.5±0.5^cd^	2.5±0.5^def^	1.6±0.5^f^
16		*α*-amorphene	1471		3.1±1.0^a^	1.2±0.3^bc^	0.8±0.1^d^	–		1.6±0.3^ab^	1.0±0.2^cd^	1.0±0.0^c^	0.6±0.2^d^
17		germacrene D	1479		–	–	–	0.4±0.0^b^		–	0.3±0.0^c^	0.5±0.0^a^	0.4±0.1^ab^
18		bicyclogermacrene	1497		4.3±0.6^ab^	3.6±0.3^b^	3.8±0.2^ab^	4.3±0.3^a^		4.0 ±0.5^ab^	3.8±0.2^ab^	5.0±0.7^a^	4.9±0.7^ab^
19		*γ*-cadinene	1513		0.8±0.2^b^	1.0±0.2^ab^	1.1±0.1^ab^	1.4±0.2^a^		1.1±0.2^ab^	1.2±0.1^ab^	1.6±0.6^ab^	1.5±0.5^ab^
20		*δ*-cadinene	1521		–	0.5±0.0^b^	0.5±0.3^b^	1.0±0.4^ab^		–	0.6±0.1^b^	1.3±0.3^a^	1.0±0.3^ab^
21		*cis*-nerolidol	1529		1.3±0.3^c^	1.3±0.1^c^	1.6±0.2^bc^	2.1±0.2^a^		1.4±0.2^c^	1.8±0.3^b^	2.5±0.1^a^	2.5±0.4^a^
22		*epi*-*α*-cadinol	1633		–	–	0.4±0.1^c^	0.6±0.1^bc^		–	0.5±0.1^c^	0.7±0.1^ab^	0.7±0.1^a^
23		*α*-cadinol	1657		2.4±0.6^d^	2.5±0.2^d^	3.2±0.6^cd^	4.2±0.5^ab^		2.6±0.7^d^	3.4±0.8^bcd^	5.1±0.8^a^	5.1±1.0^ab^
		TOTAL [%]^3^			99.6±0.5	100.0±0.0	99.7±0.5	98.4±0.6		99.6±0.7	99.2±0.8	98.6±0.6	97.8±1.0
		VOC content [%]^4^			0.4±0.1^d^	0.6±0.1^ab^	0.7±0.2^ab^	1.1±0.4^a^		0.5±0.2^bcd^	0.7±0.3^abcd^	0.8±0.2^abcd^	0.9±0.2^ab^
		VOC content [mg total LDM^-1^]^5^						15.6±1.2^b^					17.8±2.0^a^

RI, retention index; DAS, days after sowing; bold compounds represent the major volatile compounds of the cultivar; different letters within a row indicate significant differences at p ≤ 0.05. **^1^** Presented are mean RIs of samples under GC-FID conditions on an HP-5MS column relative to a series of n-alkanes. **^2^** Presented are mean percentages ± SD of volatile organic compounds of all analyzed basil leaf extracts (n = 8 leaf extracts per light treatment and DAS). **^3^** Percentage [%] ± SD represents total of listed volatile organic compounds. (Traces of eight identified compounds (namely β-pinene (RI 982), limonene (RI 1033), cis-linalool oxide (RI 1071), borneol (RI 1174), α-terpineol (RI 1196), chavicol (RI 1253), cis-methyl cinnamate (RI 1293), viridiflorol (RI 1596) and traces of two unidentified compounds (RI 1569, RI 1574) are excluded from the table.) **^4^** Presented are average VOC contents ± SD in percent [%] per gram of leaf dry matter (w/w) from four independent experimental replications per light treatment. **^5^** Presented are calculated average total VOC contents ± SD in mg total LDM^-1^ (leaf dry matter) across four spatial replications at harvest (35 DAS) per basil pot.

The corrected **Table 5** Relative abundance of major volatile organic compounds identified in the leaves of *O. basilicum* L. cv. ‘Cinnamon’ as affected by LED light intensity treatments over time and its caption appear below.

In the published article, there was an error in **Table 6** **Relative abundance of major volatile organic compounds identified in the leaves of *O. basilicum* L. cv. ‘Cinnamon’ as affected by LED light intensity treatments over time**. **Table 6** erroneously shows the compounds of ‘Cinnamon’ but has to show the compounds of cv. ‘Dark Opal’ to match the references in the text and to stay in chronological order.

**Table 6:** Relative abundance of major volatile organic compounds identified in the leaves of *O. basilicum* L. cv. ‘Dark Opal’ as affected by LED light intensity treatments over time.

No.	Compound	RI^1^	I_Low_				I_High_				
			DAS 14	DAS 21	DAS 28	DAS 35	DAS 14		DAS 21	DAS 28	DAS 35
			Percent (%) of total volatile organic compound composition^2^								
1	*α*-pinene	938	0.4±0.0^cd^	0.4±0.0^cd^	0.4±0.0^cd^	0.4±0.1^bcd^	0.4±0.0^cd^		0.4±0.1^cd^	0.5±0.0^ab^	0.5±0.1^abc^
2	*β*-pinene	982	0.5±0.0^c^	0.6±0.1^bc^	0.6±0.1^bc^	0.7±0.1^b^	0.7±0.0^b^		0.6±0.1^bc^	0.9±0.1^a^	0.9±0.1^a^
3	*β*-myrcene	992	–	0.4±0.1^f^	0.4±0.1^def^	0.5±0.1^bcdef^	0.5±0.0^ef^		0.5±0.1^def^	0.6±0.0^ab^	0.7±0.1^a^
4	limonene	1033	0.7±0.1^abc^	0.6±0.1^abc^	0.6±0.1^bc^	0.6±0.1^abc^	0.7±0.0^a^		0.6±0.1^c^	0.7±0.1^ab^	0.6±0.1^abc^
5	**1,8-cineole**	**1036**	**4.4±0.3^d^**	**5.2±0.5^d^**	**5.6±0.7^d^**	**6.6±0.8^bcd^**	**6.0±0.0^abcd^**		**6.3±0.8^d^**	**8.2±1.0^abcd^**	**9.5±1.1^a^**
6	terpinolene	1095	3.3±0.7^abcd^	2.6±0.3^a^	2.3±0.2^abc^	2.2±0.1^abc^	2.9±0.3^a^		2.1±0.2^bc^	2.0±0.2^bc^	1.3±0.2^d^
7	**linalool**	**1101**	**1.6±0.7^e^**	**2.7±0.4^e^**	**4.5±0.8^d^**	**8.8±1.5^c^**	**5.4±0.4^d^**		**8.3±1.2^c^**	**16.0±2.3^b^**	**28.4±5.0^a^**
8	*α*-terpineol	1197	–	0.3±0.0^f^	0.4±0.0^e^	0.5±0.0^cd^	0.5±0.0^de^		0.6±0.1^c^	0.7±0.1^b^	1.0±0.1^a^
9	nerol	1227	0.9±0.1^b^	0.9±0.2^b^	0.9±0.1^b^	0.9±0.1^b^	0.9±0.1^b^		1.0±0.1^b^	1.5±0.2^a^	1.7±0.2^a^
10	**eugenol**	**1364**	**-**	**0.6±0.2^g^**	**1.0±0.3^fg^**	**3.2±0.8^cd^**	**2.1±0.9^defg^**		**4.9±1.0^bc^**	**4.6±0.3^b^**	**9.0±2.5^a^**
11	*β*-elemene	1403	–	0.4±0.1^d^	0.3±0.2^cd^	0.3±0.2^cd^	0.3±0.0^d^		0.5±0.1^c^	0.5±0.1^bcd^	0.8±0.1^a^
12	**methyl eugenol**	**1408**	**75.7±3.2^ab^**	**74.2±1.7^a^**	**72.3±2.2^a^**	**63.0±3.9^cd^**	**66.3±1.5^bc^**		**61.0±2.7^d^**	**48.4±3.8^f^**	**25.2±4.3^g^**
13	*α-trans*-bergamotene	1437	1.9±0.1^a^	1.7±0.1^bc^	1.7±0.1^bc^	1.7±0.1^abc^	1.5±0.1^c^		1.7±0.0^abc^	1.7±0.1^abc^	1.9±0.2^ab^
14	aromadendrene	1446	0.4±0.1^bcd^	0.3±0.0^f^	0.3±0.0^ef^	0.3±0.0^def^	0.5±0.1^ab^		0.4±0.1^bcd^	0.4±0.1^cde^	0.4±0.1^bcd^
15	*α*-humulene	1451	0.4±0.0^de^	0.3±0.0^ef^	0.3±0.0^ef^	0.4±0.0^cde^	0.2±0.0^f^		0.4±0.0^e^	0.5±0.1^bc^	0.8±0.1^a^
16	*β*-farnesene	1461	4.4±0.8^ab^	3.2±0.3^ab^	3.2±0.2^b^	3.4±0.3^ab^	4.0±0.5^ab^		3.7±0.2^a^	3.9±0.4^a^	3.3±0.3^ab^
17	*α*-amorphene	1471	0.8±0.1^a^	0.5±0.0^cd^	0.5±0.0^de^	0.5±0.0^e^	0.6±0.0^bcd^		0.5±0.0^bcde^	0.6±0.1^bcde^	0.6±0.0^bcd^
18	bicyclogermacrene	1498	1.8±0.2^f^	1.8±0.1^f^	2.0±0.1^cdef^	2.1±0.1^bc^	1.9±0.1^cdef^		2.1±0.2^bcd^	2.3±0.2^b^	3.1±0.2^a^
19	*γ*-cadinene	1513	0.5±0.0^e^	0.5±0.0^e^	0.7±0.1^d^	1.0±0.1^b^	0.8±0.1^cd^		0.9±0.1^bc^	1.2±0.2^abc^	1.3±0.2^a^
20	*δ*-cadinene	1521	–	0.1±0.1^f^	0.4±0.0^e^	0.6±0.0^bc^	0.3±0.0^f^		0.5±0.1^de^	0.8±0.1^b^	1.4±0.1^a^
21	*cis*-nerolidol	1529	0.6±0.0^fg^	0.6±0.1^fg^	0.6±0.1^efg^	0.7±0.0^de^	0.6±0.0^g^		0.7±0.0^de^	0.8±0.1^bcd^	1.2±0.1^a^
22	*α*-cadinol	1657	1.1±0.1^fg^	1.1±0.1^g^	1.2±0.2^efg^	1.1±0.0^g^	1.1±0.0^g^		1.3±0.1^def^	1.5±0.2^bcde^	2.3±0.1^a^
	TOTAL [%]^3^		99.4±0.4	99.4±0.2	99.4±0.1	99.1±0.3	98.8±0.3		98.3±0.4	98.8±0.4	98.3±0.3
	VOC content [%]^4^		0.9±0.0^bcd^	1.2±0.2^ab^	0.9±0.2^cd^	0.8±0.1^d^	1.5±0.3^a^		1.1±0.2^abc^	0.8±0.2^d^	0.8±0.2^d^
	VOC content [mg total LDM^-1^]^5^					2.6±0.2^b^					8.5±0.6^a^

RI, retention index; DAS, days after sowing; bold compounds represent the major volatile compounds of the cultivar; different letters within a row indicate significant differences at p ≤ 0.05. **^1^** Presented are mean RIs of samples under GC-FID conditions on an HP-5MS column relative to a series of n-alkanes. **^2^** Presented are mean percentages ± SD of volatile organic compounds of all analyzed basil leaf extracts (n = 8 leaf extracts per light treatment and DAS). **^3^** Percentage [%] ± SD represents total of listed volatile organic compounds. (Traces of ten identified compounds (namely sabinene (RI 978), cis-linalool oxide (RI 1071), borneol (RI 1174), methyl chavicol (RI 1203), chavicol (RI 1256), β-cubebene (RI 1384), trans-methyl cinnamate (RI 1390), germacrene D (RI 1479), viridiflorol (RI 1596), epi-α-cadinol (RI 1633) and traces of three unidentified compounds (RI 1015, RI 1120, RI 1548) are excluded from the table.) **^4^** Presented are average VOC contents ± SD in percent [%] per gram of leaf dry matter (w/w) from four independent experimental replications per light treatment. **^5^** Presented are calculated average total VOC contents ± SD in mg total LDM^-1^ (leaf dry matter) across four spatial replications at harvest (35 DAS) per basil pot.

The corrected **Table 6** Relative abundance of major volatile organic compounds identified in the leaves of *O. basilicum* L. cv. ‘Dark Opal’ as affected by LED light intensity treatments over time and its caption appear below (which remain unaffected).

In the published article, **Figure 4** is not referenced correctly in the text.

A correction has been made to **Results and Discussion**, *I_High_ induced light avoidance responses in green basil cultivars while purple basil remained ‘light-tolerant’*, paragraph 1.

This sentence previously stated:

“As evident in **Figure 5**, undesirable morphological and anatomical leaf adaptations to I_High_ became clearly visible at the top canopy of the green-leafed cultivars cv. Anise, cv. Cinnamon and cv. Thai Magic during the last week of the trial period when PFDs exceeded an average of 300 µmol m^-2^ s^-1^ at canopy level (**Table 2**).”

The corrected sentence appears below:

“As evident in **Figure 4**, undesirable morphological and anatomical leaf adaptations to I_High_ became clearly visible at the top canopy of the green-leafed cultivars cv. Anise, cv. Cinnamon and cv. Thai Magic during the last week of the trial period when PFDs exceeded an average of 300 µmol m^-2^ s^-1^ at canopy level (**Table 2**).”

A correction has been made to **Results and Discussion**, *I_Low_ resulted in beneficial acclimation responses in green basil cultivars while purple basil performed insufficiently*, paragraph 1.

This sentence previously stated:

“In contrast to the undesirable light-avoiding leaf adaptations and flower initiations detected under I_High_ (section 3.3), the light-orientated leaves of the green cultivars cv. Anise, cv. Cinnamon and cv. Thai Magic were fully expanded, thin and displayed typical green leaf colors under I_Low_ (**Figure 5**).”

The corrected sentence appears below:

“In contrast to the undesirable light-avoiding leaf adaptations and flower initiations detected under I_High_ (section 3.3), the light-orientated leaves of the green cultivars cv. Anise, cv. Cinnamon and cv. Thai Magic were fully expanded, thin and displayed typical green leaf colors under I_Low_ (**Figure 4**).”

A correction has been made to **Results and Discussion**, *I_Low_ resulted in beneficial acclimation responses in green basil cultivars while purple basil performed insufficiently*, paragraph 2.

This sentence previously stated:

“The greater leaf sizes, the evenly distributed chloroplasts across the leaves of cv. Anise,

cv. Cinnamon and cv. Thai Magic (**Figure 5**) as well as the fact that marketability criteria were reached just 3-4 days later under I_Low_ (as described in section 3.2) are robust signs that very similar (beneficial) low light acclimation responses took place in the green cultivars investigated in our study.”

The corrected sentence appears below:

“The greater leaf sizes, the evenly distributed chloroplasts across the leaves of cv. Anise,

cv. Cinnamon and cv. Thai Magic (**Figure 4**) as well as the fact that marketability criteria were reached just 3-4 days later under I_Low_ (as described in section 3.2) are robust signs that very similar (beneficial) low light acclimation responses took place in the green cultivars investigated in our study.”

In the published article, **Figure 4C** is not referenced correctly in the text.

A correction has been made to **Results and Discussion**, *I_Low_ resulted in beneficial acclimation responses in green basil cultivars while purple basil performed insufficiently*, paragraph 3.

This sentence previously stated:

“To the contrary, the comparatively small leaf sizes (as evident in **Figure 5C**) and the slow development of cv. Dark Opal under I_Low_ (as evident by the great differences in plant height, number of developed leaf and branch pairs and thus biomass yields (**Table 2** and **Figures 4C, G, K, O**) in comparison to I_High_ show clearly, that cv. Dark Opal was not able to adjust as well as its green basil counterparts to the given low light conditions.”

The corrected sentence appears below:

“To the contrary, the comparatively small leaf sizes (as evident in **Figure 4C**) and the slow development of cv. Dark Opal under I_Low_ (as evident by the great differences in plant height, number of developed leaf and branch pairs and thus biomass yields (**Table 2**, **Figures 4C, G, K, O**) in comparison to I_High_ show clearly, that cv. Dark Opal was not able to adjust as well as its green basil counterparts to the given low light conditions.”

In the published article, **Figures 5A-H** are not referenced correctly in the text.

A correction has been made to **Results and Discussion**, *Cultivar-dependent light intensity requirements under increasing light intensity conditions*, paragraph 6.

This sentence previously stated:

“However, with the high-quality market-ready basils within 30-37 DAS (**Figures 4A–H**) detected under I_Low_ under increasing light intensities (between ~ 100 and 200 µmol m^-2^ s^-1^ (**Table 2**)) mostly below the lowest recommended constant light intensity of 180 µmol m^-2^ s^-1^ as recently reviewed by Sipos et al. (2021), the increasing light conditions applied in this study show that even with lower than previously assumed light requirements, high-quality basil productions under artificial light conditions are possible.”

The corrected sentence appears below:

“However, with the high-quality market-ready basils within 30-37 DAS (**Figures 5A–H**) detected under I_Low_ under increasing light intensities (between ~ 100 and 200 µmol m^-2^ s^-1^ (**Table 2**)) mostly below the lowest recommended constant light intensity of 180 µmol m^-2^ s^-1^ as recently reviewed by Sipos et al. (2021), the increasing light conditions applied in this study show that even with lower than previously assumed light requirements, high-quality basil productions under artificial light conditions are possible.”

In the published article, **Figures 5Q**-2 are not referenced correctly in the text.

A correction has been made to **Results and Discussion**, *Cultivar-specific VOC concentrations change over time*, paragraph 8.

This sentence previously stated:

“Different VOC accumulation patterns were observed between the investigated basil cultivars (**Figures 4Q–S**).”

The corrected sentence appears below:

“Different VOC accumulation patterns were observed between the investigated basil cultivars (**Figures 5Q–S**).”

A correction has been made to **Results and Discussion**, *Cultivar-specific VOC concentrations change over time*, paragraph 9.

This sentence previously stated:

“Within each cultivar, VOC concentrations generally did not differ between I_Low_ and I_High_ during each investigated time point, however, exceptions were observed in the cultivars ‘Dark Opal’ and ‘Thai Magic’ (**Figures 4Q–S**).”

The corrected sentence appears below:

“Within each cultivar, VOC concentrations generally did not differ between I_Low_ and I_High_ during each investigated time point, however, exceptions were observed in the cultivars ‘Dark Opal’ and ‘Thai Magic’ (**Figures 5Q–S**).”

In the published article, **Figure 5S** is not referenced correctly in the text.

A correction has been made to **Results and Discussion**, *Cultivar-specific VOC concentrations change over time*, paragraph 9.

This sentence previously stated:

“The significantly lower VOC concentration detected under I_Low_ in comparison to I_High_ at 14 DAS in ‘Dark Opal’ (**Figure 4S**) may be ascribed to the cultivars’ limited photosynthetic capabilities under low light conditions as discussed before in section 3.4 and hence, a presumably lower availability of photosynthates for secondary metabolism.”

The corrected sentence appears below:

“The significantly lower VOC concentration detected under I_Low_ in comparison to I_High_ at 14 DAS in ‘Dark Opa’ (**Figure 5S**) may be ascribed to the cultivars’ limited photosynthetic capabilities under low light conditions as discussed before in section 3.4 and hence, a presumably lower availability of photosynthates for secondary metabolism.”

In the published article, **Figures 5T** and **4D** are not referenced correctly in the text.

A correction has been made to **Results and Discussion**, *Cultivar-specific VOC concentrations change over time*, paragraph 9.

This sentence previously stated:

“The slightly increased VOC concentration observed under I_Low_ in comparison to I_High_ at 35 DAS in cv. Thai Magic (**Figure 4T**) may indicate that this cultivar reaches its maximum VOC accumulation before flower buds arise (**Figure 5D**) after which VOC concentrations decrease – an accumulation pattern that has previously been described for the *Ocimum basilicum* cultivars ‘Rit-Sat’ and ‘Lengyel’ (Szabó & Bernáth, 2002) as well as for *Ocimum ciliatum* (Moghaddam et al., 2015).”

The corrected sentence appears below:

“The slightly increased VOC concentration observed under I_Low_ in comparison to I_High_ at 35 DAS in cv. Thai Magic (**Figure 5T**) may indicate that this cultivar reaches its maximum VOC accumulation before flower buds arise (**Figure 4D**) after which VOC concentrations decrease – an accumulation pattern that has previously been described for the *Ocimum basilicum* cultivars ‘Rit-Sat’ and ‘Lengyel’ (Szabó & Bernáth, 2002) as well as for *Ocimum ciliatum* (Moghaddam et al., 2015).”

In the published article, **Figures 5U–X** are not referenced correctly in the text.

A correction has been made to Results and Discussion, *Cultivar-specific VOC concentrations change over time*, paragraph 10.

This sentence previously stated:

“When comparing total VOC yields produced at the end of the experiment (35 DAS), all cultivars had accumulated substantially greater VOC quantities under I_High_ than basils of the same age under I_Low_ due to the cultivars’ overall greater leaf biomasses accumulated under I_High_ (**Table 2** and **Figures 4U–X**).”

The corrected sentence appears below:

“When comparing total VOC yields produced at the end of the experiment (35 DAS), all cultivars had accumulated substantially greater VOC quantities under I_High_ than basils of the same age under I_Low_ due to the cultivars’ overall greater leaf biomasses accumulated under I_High_ (**Table 2** and **Figures 5U–X**).”

In the published article, there was an error in the legend for **Table 9 Energy consumption at marketability of four *Ocimum basilicum* L. cultivars grown under two different light intensities as published**. **Figures 4A–H** are incorrectly referenced.

The legend previously stated:

“DAS = days after sowing; FW = fresh weight; I_High_ = High light intensity; I_Low_ = Low light intensity; na = not available. ^1^ Marketability is defined as plant height ≥ 15 cm and/or number of leaf pairs ≥ 4 [by our cooperation partners Oderbruch Müller, an organic nursery in Bad Freienwalde, Germany]; see also **Figures 4A–H**. ^2^ Basil fresh weights [g pot^-1^] at marketability were calculated using each cultivars’ polynomial biomass rates under each light treatment

(R^2^ > 0.99).”

The corrected legend appears below:

“DAS = days after sowing; FW = fresh weight; I_High_ = High light intensity; I_Low_ = Low light intensity; na = not available. ^1^ Marketability is defined as plant height ≥ 15 cm and/or number of leaf pairs ≥ 4 [by our cooperation partners Oderbruch Müller, an organic nursery in Bad Freienwalde, Germany]; see also **Figures 5A–H**. ^2^ Basil fresh weights [g pot^-1^] at marketability were calculated using each cultivars’ polynomial biomass rates under each light treatment

(R^2^ > 0.99).”

The authors apologize for these errors and state that this does not change the scientific conclusions of the article in any way. The original article has been updated.

